# Response of ornamental plants to salinity: impact on species-specific growth, visual quality, photosynthetic parameters, and ion uptake

**DOI:** 10.3389/fpls.2025.1611767

**Published:** 2025-07-30

**Authors:** Zirui Wang, Shital Poudyal, Kelly Kopp, Yunhua Zhang

**Affiliations:** ^1^ Department of Resources & Environment, Anhui Agriculture University, Hefei, Anhui, China; ^2^ Department of Plants, Soils & Climate, Utah State University, Logan, UT, United States

**Keywords:** aesthetic performance, growth, leaf greenness, saline irrigation, ion imbalance, photosynthetic parameter, ornamental

## Abstract

Ornamental horticulture provides substantial economic and environmental benefits, generating billions in annual sales and contributing to urban greening. However, the increasing scarcity of freshwater resources necessitates the use of alternative irrigation sources, such as reclaimed water. Reclaimed water typically contains elevated salt levels that can induce salt stress. Salt stress adversely affects multiple plant traits. Aesthetic quality declines, manifesting as leaf tip burns, discoloration, and necrosis, reducing landscape value and commercial appeal. Growth performance, including biomass production, plant height, and leaf expansion, is limited by osmotic stress, ion toxicity, and nutrient imbalances. Salinity also reduces chlorophyll content, leading to lower leaf greenness and photosynthetic efficiency through impaired stomatal conductance and transpiration. In addition, saline water disrupts ion uptake, increasing Na^+^ and Cl^-^ accumulation and disrupting the balance of essential nutrients like K^+^ and Ca^2+^. These physiological and visual responses are species-specific. Therefore, this review synthesizes current findings on the impact of salinity stress on ornamental plants, with a focus on aesthetic value, growth performance, photosynthetic traits, and ion homeostasis. It aims to inform sustainable irrigation practices and species selection for nursery production and landscape applications using alternative water sources with salinity concerns.

## 1 Introduction

Horticulture is a specialized branch of agriculture that encompasses both the art and science of cultivating fruits, vegetables, and ornamental plants, including flowers, trees, and shrubs. One of its primary divisions is ornamental horticulture, which focuses on the production, management, and marketing of plants selected for their aesthetic value (Jaskani and Khan, 2021). The economic significance of ornamental plants in horticulture is substantial. In 2022, sales of floriculture, nursery, and greenhouse plants reached approximately $21.3 billion, representing a 31.9% increase from 2017 levels (US Department of Agriculture, National Agricultural Statistics Service, 2020).

Ornamental horticulture represents a thriving sector within agriculture, driven not only by its substantial economic returns, but also by its growing relevance in urban ecological planning. As cities expand and green infrastructure becomes increasingly prioritized, ornamental plants are recognized not just as commodities but as integral components of sustainable urban ecosystems. Research has shown that urban horticulture, including ornamental plants, can reduce carbon dioxide (CO_2_) emissions (Ohyama et al., 2008). For example, in United States, urban green spaces sequester around 22.8 million tons of annually, equivalent to 83.6 million tons of CO_2_ (Nowak and Crane, 2002; Zhang et al., 2024). Furthermore, ornamental plants contribute to humidity and temperature regulation, abating the urban heat island (Francini et al., 2008). They also enhance resource efficiency by conserving water, fertilizers, and energy inputs (Ohyama et al., 2008).

However, the growing impact of climate change is beginning to challenge the ability of these green spaces to flourish and has the impact has been obvious especially in parts of Africa, South Asia, and the American Southwest (Neha et al., 2025; Shrivastava and Kumar, 2014). Rising temperature and reduced precipitation, increases evapotranspiration and can bring salt and other minerals to soil surface thus, increasing soil salinity (Cook et al., 2018; Rengasamy, 2006). Climate change is also reducing frequency and increasing intensity of precipitation which causes intermittent drought and flooding conditions and is detrimental to plant growth (Thakre and Bisen, 2023). For instance, floriculture in Kenya is grappling with prolonged drought due to climate change (Neha et al., 2025). United States has experienced widespread drought conditions in recent two decades, with 54.8% of the country affected in 2012, marking one of the most extensive drought periods (US Drought Monitor, 2025). In response, recycled or reclaimed water, defined as water that has already been used, has emerged as a critical alternative water resource for irrigation, especially in arid and semi-arid regions (Chaudhary et al., 2019; Niu and Cabrera, 2010). This approach addresses water scarcity by repurposing wastewater for landscape use. For instance, in Florida, reclaimed water irrigates 56,000 acres of golf courses (approximately 227 million square meters), 201,465 residences, 572 parks, and 251 schools in 2005 (Haering et al., 2009). However, significant concerns exist regarding the environmental impacts of reclaimed water, with salinity representing one of the most severe challenges (Nackley et al., 2015).

Cabrera et al. (2018) found that municipal reclaimed water in Texas contained sodium (Na^+^) and chloride (Cl^-^) concentrations of 90–280 mg·L^-1^ and 135–340 mg·L^-1^, respectively, exceeding the recommended thresholds for landscape irrigation water (70 mg·L^-1^ and 110 mg·L^-1^, respectively) (**Table 1**). Electrical conductivity (EC), measured in deciSiemens per meter (dS·m^-1^), serves as an indicator of dissolved salts and ions in water (Ezlit et al., 2010). Bauder et al. (2011) and Fipps (2003) reported that water with EC below 0.75 dS·m^-1^ is generally suitable for irrigation, while with EC above 2 dS·m^-1^ can pose risks to plants and soil, whereas EC in Texas reclaimed water ranged from 0.9 to 1.9 dS·m^-1^ (Cabrera et al., 2018). Under suboptimal management, low-quality reclaimed water can exacerbate soil salinity issues. As shown in **Table 2**, a study by Huang et al. (2011) demonstrated the progressive soil EC increase during 120 days of irrigation with water at EC of 1.2, 2.6, and 7.0 dS·m^-1^. When irrigating water at an EC of 2.6 and 7.0 dS·m^-1^, soil EC rose significantly in both 0–30 cm (to 5.0 and 7.6 dS·m^-1^, respectively) and 30–60 cm soil layers (to 4.3 and 6.3 dS·m^-1^, respectively). Conversely, irrigation with water at an EC of 1.2 dS·m^-1^ maintained relatively stable soil EC, with the 0–30 cm layer around 3.2 dS·m^-1^ and a slight decrease to 3.0 dS·m^-1^ in 30–60 cm layer. Generally, soil with EC levels exceeding 4 dS·m^-1^ is classified as saline soil (Zaman et al., 2018; Singh, 2022). These findings suggest that even moderately saline water can lead to soil salinization over time. Therefore, the use of reclaimed water, particularly under poor management, should be carefully regulated to prevent long-term soil degradation and salinity-induced stress in landscape plants.

**Table 1:** Reclaimed water quality and recommended values for sodium, chlorine and electrical conductivity.

Water quality	Observed values	Recommend threshold	Reference
Sodium	90–280 mg·L^-1^	70 mg·L^-1^	Bauder et al. (2011); Cabrera et al. (2018), and Fipps (2003)
Chloride	135–340 mg·L^-1^	110 mg·L^-1^	
Electricity conductivity (EC)	0.9-1.9 dS·m^-1^	0.75 dS·m^-1^	

**Table 2:** Impacts of saline irrigation on soil electricity conductivity (EC).

Treatment (dS·m^-1^, 120 days)	Soil depth (cm)	Soil EC (dS·m^-1^)	Saline soil threshold (dS·m^-1^)	Reference
1.2	0-30	3.2	4	Huang et al. (2011)
	30-60	3.0		
2.6	0-30	5.0		
	30-60	4.3		
7.0	0-30	7.6		
	30-60	6.3		

Soil salinity can arise not only from anthropogenic sources but from natural sources as well. Globally, 25%-30% of cultivated and irrigated lands are saline and commercially unproductive due to natural cause (Zaman et al., 2018). Natural causes include mineral weathering, salt-rich groundwater, high evaporation, and volcanic activity (Majeed and Muhammad, 2019; Stavi et al., 2021). Beyond poor irrigation practices, human-induced soil salinization can result from imbalanced fertilization, soil degradation, inadequate drainage, deforestation, and mining activities (Majeed and Muhammad, 2019; Tanji, 2002). Under challenging saline conditions, nature demonstrates resilience: halophytes, which are the plant species that can grow at salinities over 250 mM sodium chloride (NaCl, ~ 25 dS·m^-1^) (Tuteja, 2007). The salt-resistance mechanisms of halophyte’s are often classified as salt tolerance and salt avoidance. Salt tolerance enables plants to maintain protoplasmic viability while accumulating ions inside cells, whereas salt avoidance involves minimizing salt concentrations in potentially toxic plant parts (Aslam et al., 2011). However, most plants species remain sensitive to salt stress, with only around 2% of angiosperm species being halophytes (Turcios et al., 2021). For example, although the families Asteraceae, Fabaceae, and Poaceae include a considerable number of halophytes, these account for less than 5% of their total species (Aslam et al., 2011; Turcios et al., 2021). Consequently, it is critical to assess the responses of ornamental species under saline conditions to determine their adaptation, identify salt-tolerant candidates for landscape use, and guide appropriate plant selection and management practices in salt-affected areas.

**Figure 1:**
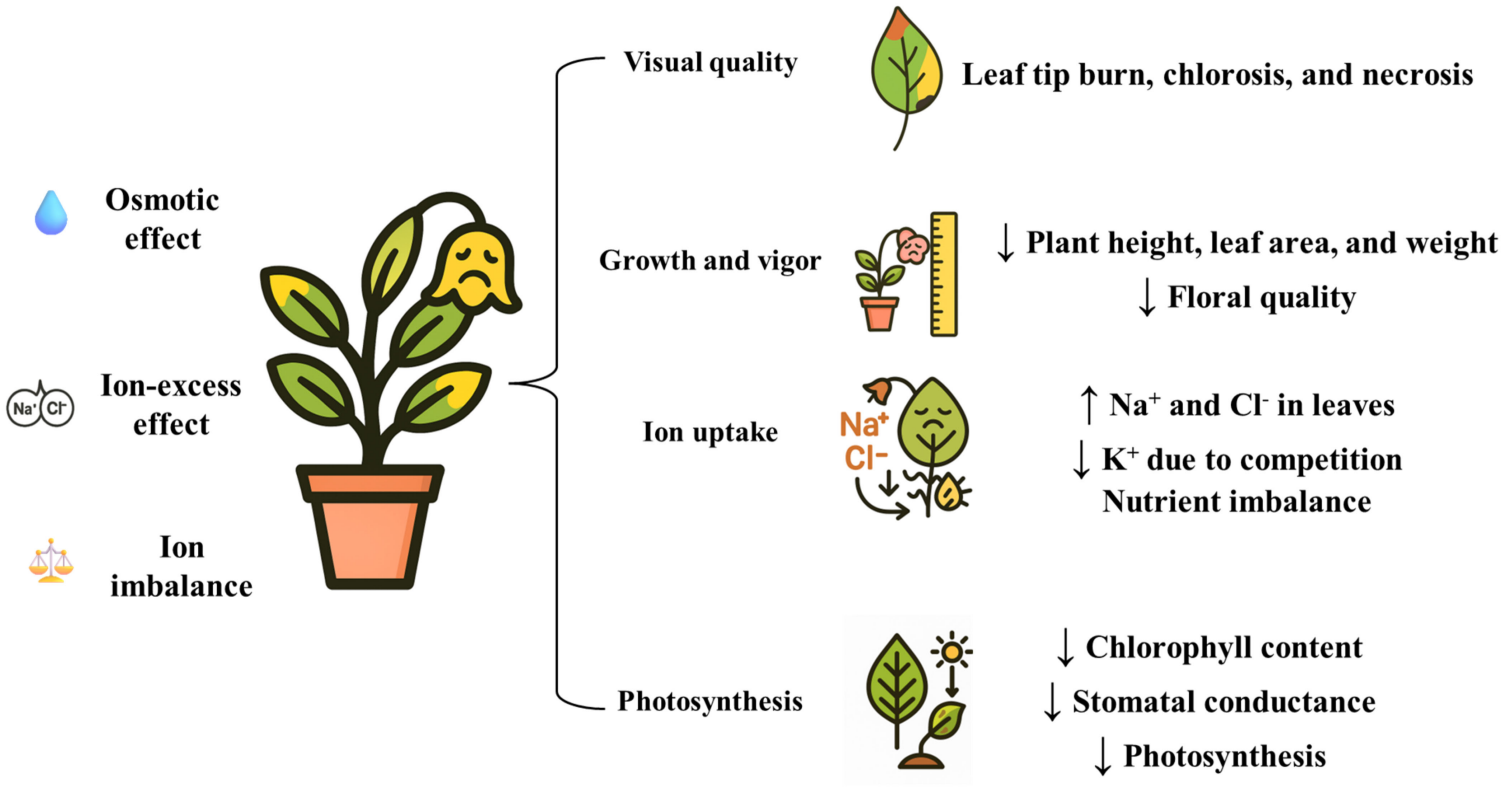
Three mechanisms of salt stress impacting plant.

Saline soil and irrigation water severely impair plant performance through three critical mechanisms (**Figure 1**). Firstly, the osmotic effect reduces water potential, preventing plant water absorption as salt in the soil solution creates barriers to water uptake (Atta et al., 2023; Singh et al., 2014). Secondly, the ion-excess effect occurs when excessive salt enters the transpiration stream, damaging leaf cells and further compromising plant growth (Parihar et al., 2015). Thirdly, excessive Na^+^ levels can disrupt ion uptake through cation competition, potentially reducing the absorption of critical ions like potassium (K^+^), calcium (Ca^2+^), and magnesium (Mg^2+^), which may lead to nutrient deficiencies (Atta et al., 2023). These detrimental effects induce significant morphological and physiological responses of plants. Initially, reduced leaf and shoot growth are the earliest response when non-halophytes plants are exposed to salinity, from water deficit or specific salt toxicity (Munns and Termaat, 1986; Negrão et al., 2017). Empirical evidence supports these effects, as severe reductions in growth and biomass have been observed in species like *Nasturtium officinale* and coleus under saline irrigation (Kaddour et al., 2013; Kotagiri and Kolluru, 2017).

Another salt-induced effect is the reduction in aesthetic value, a critical component of ornamental plants that should be taken into consideration (Cassaniti et al., 2013). The visual performance of plants under saline stress varies significantly among species. Some, like *Albizia julibrissin* and *Sophora japonica*, showed no visible salt damage at EC 5 dS·m^-1^, while others, such as Zinnia spp., exhibited significant flower reduction and leaf necrosis even under lower salinity levels of 2.6-4.5 dS·m^-1^ (Devitt and Morris, 1987; Paudel and Sun, 2022; Villarino and Mattson, 2011). Saline stress negatively impacts leaf greenness, which researchers measure through chlorophyll content, Soil Plant Analysis Development (SPAD) index, or maximum quantum yield of photosystem II (*F*_v_/*F*_m_) (Negrão et al., 2017). Studies across various species demonstrate diverse responses to salt exposure. For example, while Penstemon spp. and *Japanese* sp*iraea* showed significant declines in SPAD values, the leaf greenness of *Ageratum conyzoides*, *Nasturtium officinale*, and Osteospermum spp. did not affected by saline irrigation (Paudel and Sun, 2024; Pavlova et al., 2021; Sun et al., 2012; Valdés et al., 2015; Wang et al., 2019a). Reduced stomatal conductance was also commonly observed and, along with declined leaf greenness, contributed to the suppression of photosynthesis. This photosynthetic inhibition has been documented in multiple species, including *Cercocarpus ledifolius*, *Euphorbia milii*, *Glaucium flavum*, Rosa spp.*, Sophora secundiflora*, and *Viburnum* (Cai et al., 2014a; Chen et al., 2020; Cambrollé et al., 2011; Niu et al., 2010a; Paudel and Sun, 2022; Santos et al., 2022).

## 2 Research methodology

This goal of this review is to identify ornamental species of both economic and horticultural importance for evaluating salinity tolerance. Species selection for this review was initially guided by the 2019 Census of Horticultural Specialties, published by the USDA National Agricultural Statistics Service (NASS), which provides national-level production and sales data. This census served as the foundation to prioritize ornamental species that are widely cultivated, economically significant, and commonly used in nursery production and landscape design. Plus, peer-reviewed publications were chosen to evaluate physiological and morphological responses of more ornamental species under saline stress, with a focus on research conducted by Texas A&M AgriLife Research Center and Utah State University. These institutions, showing significant contributions to salinity studies, provided robust experimental data through greenhouse and field research involving a wide range of ornamental species. In addition to peer-reviewed journal platforms, relevant extension bulletins and technical reports were also included to supplement applied horticultural insights.

Scientific literature was sourced using journal databases and publisher platforms, including ScienceDirect, SpringerLink, and Wiley Online Library, as well as search tools such as Google Scholar and Multidisciplinary Digital Publishing Institute (MDPI) open-access portal. The search strategy incorporated combinations of keywords such as “salinity tolerance”, “ornamental plants”, “visual quality”, “growth”, “photosynthesis”, and “ion uptake”, along with specific plant names. Inclusion criteria were defined to ensure consistency: (1) saline treatments must be clearly defined and controlled; (2) results must include clear description or quantitative data on aesthetic performance, growth parameters, photosynthetic performance, or leaf ion content. Studies lacking such data were excluded; (3) only studies that induced saline stress through saline irrigation water were considered. This criterion ensured consistency across experiments, as salinity was applied in a controlled manner using solutions with specified EC levels.

Unlike general reviews that broadly examine morphological, biochemical, and physiological traits across a random selection of plants, this review takes a more focused and practical approach. It aims to identify salt-tolerant and salt-sensitive ornamental species based on their widespread cultivation and economic value. By assessing species-specific responses to saline irrigation, this review evaluates key traits such as aesthetic performance, growth characteristics, photosynthetic activity, and ion uptake. The goal is to provide a practical and evidence-based list of ornamental plants suitable for nursery production and landscape use under varying levels of salinity. In doing so, the review offers actionable insights and management recommendations that support the selection and use of salt-tolerant ornamentals, promoting water-wise landscaping practices in arid and semi-arid environments.

## 3 The effects of salt stress on ornamental plants

The severity of salt stress experienced by ornamental plants is determined by several key factors, including taxa, saline level, and duration of exposure. Firstly, different taxa or even cultivars within a species vary in their responses to salt stress due to genetic differences in ion exclusion, compartmentalization, and osmotic adjustment mechanisms (Guo et al., 2022; Niu et al., 2013; Sun et al., 2020). Secondly, saline level, which can be quantified using EC of irrigation water, directly affects plant performance. High EC levels are typically associated with higher salt concentration and greater osmotic stress and ion toxicity, which can impair visual quality, stunt growth, and disrupt ionic balance (Guo et al., 2022; Sun et al., 2020). Thirdly, prolonged duration of exposure to saline stress allows salts to accumulate in the root zone and plant tissues, intensifying injury symptoms over time (Niu et al., 2013; Sun et al., 2020). In addition, environmental factors, such as temperature and humidity, plant developmental stage, growing season and substrate characteristics can further influence plant responses to salt stress.

### 3.1 Visual quality/aesthetic performance

To assess the salt tolerance of ornamental plants, it is crucial to evaluate their visual quality since ornamentals are primarily valued for their aesthetic nature and display values (Kumar, 2023; De Oliveira et al., 2018). Sensory analysis stands out as a method to gauge aesthetic performance, capturing plant quality through overall appearance and consumer preference. De Oliveira et al. (2018) conducted sensory analysis by 352 volunteers where they rated four ornamental species on a scale from 1 (extremely disliked) to 9 (extremely liked), followed by consumer purchasing preferences. In addition, Santagostini et al. (2014) utilized trained assessors to rank photos of rosebush varieties based on flower quantity, open flower area, and flowering quality for sensory evaluation. Eye-tracking offers another approach to assess natural aesthetic values, revealing visual attention and movement patterns which were used to rate ornamental plants in urban green spaces (Scott et al., 2020; Zheng et al., 2022).

While sensory analysis provides a rapid and non-destructive means to diagnose salt-induced leaf damage, it requires substantial labor. Under salt stress, toxicity manifests when toxic ions like Na^+^ and Cl^-^ accumulate excessively in the rhizosphere (Cassaniti et al., 2013). Slight bronzing and yellowing at leaf tips are initial symptoms of Cl^-^ toxicity, which may progress to tip death and necrosis. In contrast, Na^+^ toxicity typically begins with marginal yellowing and advances to progressive necrosis (Cassaniti et al., 2013; Saeed et al., 2020). Researchers have observed chlorosis, browning, wilting, and foliage death in various ornamental species under salt stress conditions which lower aesthetic value of the plant (Gerber et al., 2011; Niu et al., 2012b; Rauter et al., 2021; Roozbahani et al., 2020; Villarino and Mattson, 2011). Thus, foliage appearance serves as a critical criterion for assessing ornamental plant responses to salinity. Precise visual evaluation of salt injuries remains challenging without universal standardized scales. Consequently, methodologies based on percentage of foliage damage and detailed plant appearance descriptions have been developed and widely used to rate visual quality of ornamental plants (**Table 3**) (Cai et al., 2014b; Moore et al., 2019; Salachna, 2024; Sun et al., 2015b; Valdez-Aguilar et al., 2011).

**Table 3:** Some aesthetic quality rating scales for evaluating salt damage on the ornamental plants.

Rating/marks	Considered attributes	References
1 = over 50% foliar damage; 2 = moderate (25% to 50%) foliar damage; 3 = slight (less than 25%) foliage damage; 4 = good quality with little foliar damage (acceptable as landscape performance); and 5 = excellent without foliar damage	Percentage of foliage damage, including burning, discoloring, and leaf death	Cai et al., 2014b
1 = plants dead; 2 = wilted plants with significant necrosis; 3 = plants wilted with necrosis; 4 = plants with slight wilting, no necrosis; and 5 = plants not wilted and no visible necrosis	Leaf wilting and necrosis	Moore et al., 2019
1 = low attractiveness, expressed in poor foliage, growth, and habit, and insufficient tolerance to salinity stress, 2 = moderate foliar damage, 3 = slight foliage damage, 4 = good quality with minimal foliar salt damage (acceptable as landscape performance) and 5 = maximum decorative effect, expressed in even growth, attractive habits, healthy foliage, and very good tolerance to salinity	Foliage damage and overall growth	Salachna, 2024
0 = dead; 1 = severe (over 90%); 2 = moderate (50% to 90%); 3 = slight (less than 50%); 4 = good quality with minor foliar damage; and 5 = excellent with no visible foliar damage	Percentage of visible foliage damage, including leaf edge burn, necrosis, and discoloration	Sun et al., 2015a
1 (poor quality, leaf bronzing higher than 75% or dead plants) to 5 (best quality, no leaf bronzing)	Percentage of leaf bronzing and death	Valdez-Aguilar et al., 2011

While the factors that reduce aesthetic quality are generally well understood, the responses of ornamental plants to saline irrigation vary widely among species due to the unique characteristics of each plant taxon. **Supplementary Table 1** summarizes the aesthetic performance of 131 ornamental species from 34 botanical families under varying levels of saline irrigation. For instance, *Achillea millefolium* was unaffected by salinity treatments at EC 5.4 dS·m^-1^ for 103 days and EC 4 dS·m^-1^ for 10 weeks (Niu and Rodriguez, 2006a; Niu et al., 2007). In contrast, *Echinacea purpurea* exhibited unacceptable performance when irrigated at EC 2 dS·m^-1^ for 10 weeks (Niu and Rodriguez, 2006a). Furthermore, certain species demonstrated tolerance to saline irrigation in term of visual performance at EC between 10–12 dS·m^-1^ for 8–12 weeks, such as *Dicliptera suberecta*, *Gazania rigens*, *Ruellia brittoniana*, three sedum species (*Sedum telephium*, *S. reflexum*, and *S. rupestre*), and three zoysia species (*Zoysia matrella*, *Z. minima*, and *Z. japonica*) (Hooks et al., 2022; Hooks and Niu, 2019; Niu and Rodriguez, 2006b; Sun et al., 2015a; Wu et al., 2016a). Other species maintained good quality with minimal damage at EC ~5 dS·m^-1^ for 4–8 weeks but suffered severe damage (50%-90%) at EC 10 dS·m^-1^, such as *Arctostaphylos uva-ursi*, *Festuca glauca*, *Hydrangea quercifolia*, *Melampodium leucanthum*, *Perovskia atriplicifolia*, and *Tagetes lemmonii* (Paudel and Sun, 2023; Niu et al., 2020; Wu et al., 2016c; Xing et al., 2021). Conversely, some ornamental species, such as *Diervilla rivularis*, *Lantana camara*, *Physocarpus opulifolius*, *Ranunculus asiaticus*, and *Zinnia angustifolia*, were highly sensitive to saline irrigation, showing extensive salt injury even at EC 5 dS·m^-1^ or lower (Bañón et al. 2011; Chen et al. 2019a; Liu et al., 2017; Rauter et al. 2021; Villarino and Mattson 2011). In addition, different species within the same genus exhibited varied responses under identical salt treatments. For example, *Viburnum dilatatum* suffered more than 90% foliage damage at EC 5 dS·m^-1^, whereas other six viburnum species maintained high quality (Sun et al., 2020). Similarly, among rosa cultivars irrigated at EC 6.4 dS·m^-1^ for 10 weeks, ‘Belinda’s Dream,’ ‘Caldwell Pink,’ and ‘Quietness’ showed good to excellent quality with minimal foliage damage, while ‘Carefree Beauty,’ ‘Folksinger,’ and ‘Winter Sunset’ experienced more than 90% leaf burn (Niu et al., 2013).

Besides genetic differences, salinity-induced injury in ornamental plants is influenced by salinity levels. For instance, *Viburnum dentatum* and *V. nudum* exhibited good quality with minimal damage at EC 5 dS·m^-1^ for 8 weeks but suffered more than 90% foliage damage at EC 10 dS·m^-1^ (Sun et al., 2020). Similarly, three penstemon species displayed good foliage quality with minimal or slight damage at EC 5 dS·m^-1^ for 8-week irrigation yet experienced 90% or more foliage damage when the salinity increased to EC 10 dS·m^-1^ (Nepal et al., 2024; Paudel and Sun, 2024). The duration of saline irrigation is another critical factor affecting plant visual performance. For example, *Spiraea japonica* maintained good quality at EC 3 dS·m^-1^ for 8 weeks but showed 50% leaf damage after 11 weeks of treatment (Chen et al., 2019a; Wang et al., 2019a). Notably, the same species may exhibit different responses depending on the experimental period. *Hibiscus syriacus* was unaffected by EC 6.5 dS·m^-1^ for 11-week irrigation but exhibited more than 50% foliage damage at EC 5 dS·m^-1^ for 8-week treatment (Chen et al., 2019a; Liu et al., 2017). The discrepancy may be attributed to seasonal differences, as Chen et al. (2019a) conducted their experiment from Oct to Nov, whereas Liu et al. (2017) performed theirs from March to May.

### 3.2 Growth and vigor

Under salt stress, the water absorption ability is reduced, leading to water stress and toxic effects that inhibit growth (Munns and Tester, 2008; García-Caparrós and Lao, 2018). Growth measurement is a common tool used by horticulturists and landscape planners to assess the salt tolerance of ornamental plants. Evaluating plant quality typically involves traditional growth measurements, including height, flower, shoot and leaf number (Kumar, 2023). Miyamoto et al. (2004) established plant tolerance thresholds based on EC of soil saturation extract (ECe), defining critical values at which 25% foliage injury or growth reduction occurs. According to this classification, the plants species are classified into five categories: sensitive (0–3 dS·m^-1^), moderately sensitive (3–6 dS·m^-1^), moderately tolerant (6–8 dS·m^-1^), tolerant (8–10 dS·m^-1^), and highly tolerant (> 10 dS·m^-1^) (De Oliveira et al., 2018; Miyamoto et al., 2004). **Supplementary Table 2** summarizes the growth responses of 177 species from 46 botanical families under varying levels of salt stress. Although the EC treatments in **Supplementary Table 2** represent saline irrigation rather than ECe, these findings still provide valuable insights into selection of salt-tolerant plants.

Water osmotic withdrawal from enlarging cells could cause turgor pressure dropping below the stress threshold needed for cell expansion (Meloni et al., 2001). As a result, the most immediate response to saline irrigation is a decrease in the leaf expansion rate, manifested as reduced leaf area (Hassan, 2024). A substantial reduction in leaf area was observed in most species under saline irrigation in **Supplementary Table 2**. However, species such as *Ageratum conyzoides*, *Juncus effusus*, and *Leymus arenarius* showed little change in leaf area when irrigated with EC 9.7 dS·m^-1^ for 20 days, EC 5 dS·m^-1^ for 8 weeks, and EC 10 dS·m^-1^ for 18 weeks, respectively, indicating high salt tolerance (Sun et al., 2012; Sun and Palmer, 2018). For species moderately sensitive to saline irrigation, such as *Acorus gramineus*, *Albizia julibrissin*, *Aquilegia canadensis*, *Carex vulpinoidea*, *Catharanthus roseus*, *Parthenocissus quinquefolia*, *Penstemon davidsonii*, *Stachys coccinea*, and *Viburnum plicatum*, reductions in leaf area were less than 50% when irrigated with EC ranging from 2.5–10 dS·m^-1^ for 6 weeks to 4 months (Liu et al., 2017; Mohammadi Kabari et al., 2024; Nepal et al., 2024; Paudel and Sun, 2022; Sun and Palmer, 2018; Sun et al., 2020; Wu et al., 2016a, c; Xing et al., 2021). In contrast, species such as *Diervilla rivularis*, *Lobelia cardinalis*, and *Penstemon strictus* exhibited more than 90% reduction in leaf area after 8 weeks of irrigation at EC 2.5–10 dS·m^-1^ (Liu et al., 2017; Paudel and Sun, 2024; Wu et al., 2016a).

When plants are subjected to saline irrigation, another typical symptom is a reduction in height due to decreased water absorption and the toxic accumulation of salts (Hao et al., 2021; Liu et al., 2024). Therefore, species that maintain height under saline conditions are considered to be more salt-tolerant. For example, *Begonia hiemalis*, *Echinacea purpurea*, *Ipomoea tricolor*, *Juncus effusus*, and *Pelargonium graveolens* maintained height growth under irrigation with EC ranging from 4-12.9 dS·m^-1^ for 3–10 weeks (Chrysargyris et al., 2021; Niu and Rodriguez, 2006a; Mircea et al., 2023; Sun and Palmer, 2018; Villarino and Mattson, 2011). Similarly, although stunted height was observed in *Acorus gramineus*, *Anisacanthus quadrifidus*, *Dicliptera suberecta*, *Salvia farinacea*, and *Schizachyrium scoparium*, the reductions were less than 30% after saline irrigation for periods ranging from 8 weeks to 95 days at EC 10 dS·m^-1^, suggesting that these species could still be utilized in landscapes irrigated with low-quality water (Sun et al., 2015a; Xing et al., 2021; Wang et al., 2019b; Wu et al., 2016a). Meanwhile, some species exhibit high sensitivity to saline irrigation. Paudel and Sun (2024) reported a 92% reduction in height for *Penstemon barbatus* under EC 10 dS·m^-1^ irrigation for 8 weeks.

A decrease in dry weight (DW) and fresh weight (FW) has been consistently observed in all plant parts, such as leaf, shoot, and root, under salt stress, with the most noticeable reductions occurring in the aerial parts (Acosta-Motos et al., 2017; Mohammadi Kabari et al., 2024; Pavlović et al., 2019). These reductions in DW and FW are primarily due to decreases in leaf area and plant height (Acosta-Motos et al., 2017; García-Caparrós and Lao, 2018). For instance, while saline irrigation had no significant impact on the height and shoot DW of *Echinacea purpurea* and *Pelargonium graveolens*, *Viburnum pragense* exhibited a similar reduction (~56-63%) in height, leaf area, and shoot DW (Chrysargyris et al., 2021; Niu and Rodriguez, 2006a; Sun et al., 2020). Similar to leaf area and height, reductions in DW and FW vary among species under salt stress, as recorded in previous reviews. Greenway and Munns (1980) reported a 40% reduction in DW for salt-sensitive soybean plants after 14 days in 10 mM NaCl (~0.64 dS·m^-1^). In contrast, some halophytes, such as *Puccinellia peisonis*, can accumulate up to 200 mM Na^+^ (~12.9 dS·m^-1^) in their shoots without significant damage. Similar trends have been observed in ornamental species, as summarized in **Supplementary Table 2**.

In floriculture, flower number reductions due to salt stress can negatively affect crop sales, making flower responses under saline irrigation an important consideration for ornamental plant evaluation (Acosta-Motos et al., 2017). **Supplementary Table 2** indicates that flowers are generally more adversely affected by saline irrigation than leaf area and height. In several species, a 100% reduction in flower number was observed (Devitt and Morris, 1987; Don et al., 2010; Wu et al., 2016c). In addition, only two species in **Supplementary Table 2**, *Petunia hybrid* and *Portulaca grandiflora*, exhibited no significant reduction in flower number under saline irrigation (Devitt and Morris, 1987; Fornes et al., 2007). It is important to note that the number of flowers is not equivalent to their quality, as other parameters, such as color, fragrance, texture, shape, and flowering stage, are also crucial for assessing ornamental value (Hůla and Flegr, 2016; Kumar, 2023). As a result, comprehensive rating scales for flower quality are necessary. For example, Valdez-Aguilar et al. (2013, 2014) reported that Lisianthus spp. maintained remarkable flower quality under saline irrigation with EC levels below 7 dS·m^-1^. However, flower quality scales are not as commonly employed to evaluate salt tolerance, in contrast to the more widely used leaf salt damage ratings.

### 3.3 Ion uptake/plant nutrient

Na^+^ is the sixth most abundant element in the Earth’s crust, comprising approximately 2.6-2.8%, and its widespread presence contributes to Na^+^ accumulation in soils (Cramer, 2002; Kronzucker et al., 2013; Subbarao et al., 2003). Consequently, salinity has become a common abiotic stress in agriculture. While excessive Na^+^ is widely recognized as a major factor limiting plant growth in salt-affected soils, some studies have reported its beneficial effects. For instance, Na^+^ has been associated with improved growth and yield in *Avena sativa* when applied as sodium nitrate (NaNO_3_), potentially by partially replacing K^+^ in plant functions (Harmer and Benne, 1945). In addition, the halophyte *Atriplex portulacoides* exhibited enhanced growth at external Na^+^ concentrations up to 200 mol·m^-3^ (~20.2 dS·m^-1^) and maintained some growth even at higher salinity levels (Redondo-Gómez et al., 2007). Despite these potential benefits, Na^+^ toxicity remains a major constraint on plant productivity, particularly in non-halophytic species. Excess Na^+^ disrupts ion homeostasis, leading to physiological stress and reduced growth across large terrestrial areas (Blumwald et al., 2000; Munns and Tester, 2008; Kronzucker et al., 2013). NaCl is the most soluble and widespread salt contributing to soil salinity, and its detrimental effects on plants include growth inhibition and decreased productivity (Ashrafi and Rezaei Nejad, 2018; Bezerra et al., 2020; Blumwald et al., 2000; Gholamzadeh Alam et al., 2022; Munns and Tester, 2008). One of the earliest visible symptoms of Na^+^ toxicity is foliar damage, which begins in the oldest leaves and manifests as tip and marginal burn, scorch, and necrosis, which are factors that significantly diminish the ornamental value of landscape plants (Cassaniti et al., 2009, 2013; Villarino and Mattson, 2011). **Table 4** summarizes ion content responses in the leaves of 128 ornamental species across 40 botanical families under varying levels of saline irrigation. In most studies, NaCl was introduced through irrigation water to simulate saline conditions, leading to a general increase in leaf Na^+^ content. However, in some species, including *Ipomoea purpurea*, *Ranunculus acris*, *Rosa ×fortuniana*, *Sophora japonica*, and *Viburnum opulus*, Na^+^ accumulation was not significantly affected by saline irrigation (Niu et al., 2008; Mircea et al., 2023; Paudel and Sun, 2022; Sun et al., 2020; Wala et al., 2023). A particularly striking case is *Viburnum opulus*, where Na^+^ content increased 36-fold after 8 weeks of saline irrigation at EC 10 dS·m^-1^, though this increase was not statistically significant (Sun et al., 2020). Conversely, *Sophora japonica* exhibited no significant change in Na^+^ content under the same treatment, suggesting that salt-tolerant species may possess mechanisms to restrict Na^+^ uptake or transport (Paudel and Sun, 2022). Similarly, Na^+^ content for *Ipomoea purpurea* and *Ranunculus acris* suggests the presence of mechanisms that block long-distance Na^+^ transport, confining excess Na^+^ to the roots (Mircea et al., 2023; Wala et al., 2023). In addition, *Rosa ×fortuniana* has been reported to possess an effective Na^+^ exclusion mechanism, further highlighting the species-specific nature of Na^+^ regulation under saline stress (Niu et al., 2008).

**Table 4:** Effects of saline irrigation on nutrient concentrations in leaves in different ornamental species.

Botanical family	Species	Salt treatments	Ca	Cl	Na	K	References
Acanthaceae	*Anisacanthus quadrifidus*	10 dS·m^-1 i^, 8 weeks^ii^	–^iii^	↑^iv^	↑	–	Wu et al., 2016a
Acanthaceae	*Dicliptera suberecta*	10 dS·m^-1^, 8 weeks	↑	↑	↑	↓^v^	Wu et al., 2016a
Acanthaceae	*Ruellia brittoniana*	10 dS·m^-1^, 8 weeks	–	↑	↑	↓	Sun et al., 2015a
Adoxaceae	*Viburnum×burkwoodii*	10 dS·m^-1^, 8 weeks	↑	↑	↑	–	Chen et al., 2020; Sun et al., 2020
Adoxaceae	*Viburnum cassinoides*	10 dS·m^-1^, 8 weeks	↑	↑	↑	–	Sun et al., 2020
Adoxaceae	*Viburnum dentatum*	10 dS·m^-1^, 8 weeks	↑	↑	↑	↑	Sun et al., 2020
Adoxaceae	*Viburnum dilatatum*	10 dS·m^-1^, 8 weeks	↑	↑	↑	↑	Sun et al., 2020
Adoxaceae	*Viburnum×*’NCVX1’	10 dS·m^-1^, 8 weeks	↑	↑	↑	↑	Chen et al., 2020; Sun et al., 2020
Adoxaceae	*Viburnum nudum*	10 dS·m^-1^, 8 weeks	↑	↑	↑	↑	Chen et al., 2020; Sun et al., 2020
Adoxaceae	*Viburnum opulus*	10 dS·m^-1^, 8 weeks	↑	↑	–	–	Sun et al., 2020
Adoxaceae	*Viburnum plicatum*	10 dS·m^-1^, 8 weeks	↑	↑	↑	–	Sun et al., 2020
Adoxaceae	*Viburnum pragense*	10 dS·m^-1^, 8 weeks	↑	↑	↑	–	Chen et al., 2020; Sun et al., 2020
Adoxaceae	*Viburnum×rhytidophylloides*	10 dS·m^-1^, 8 weeks	↑	↑	↑	–	Chen et al., 2020; Sun et al., 2020
Adoxaceae	*Viburnum trilobum*	10 dS·m^-1^, 8 weeks	↑	↑	↑	–	Sun et al., 2020
Apocynaceae	*Catharanthus roseus*	4.7 dS·m^-1^, 4 months	↓	↑	↑	↓	Mohammadi Kabari et al., 2024
Apocynaceae	*Catharanthus roseus*	8.1 dS·m^-1^, 50 days	↓	^vi^	↑	↓	Cartmill et al., 2013
Asteraceae	*Calendula officinalis*	~4.7 dS·m^-1^, 137 days		↑	↑		Swaefy and El-Ziat, 2020
Asteraceae	*Calendula officinalis*	9.7 dS·m^-1^, 4 weeks	–	↑	↑	–	Kozminska et al., 2017
Asteraceae	*Calendula officinalis*	12.5 dS·m^-1^, 70 days	–	↑	↑	↓	Fornes et al., 2007
Asteraceae	*Chrysactinia mexicana*	10 dS·m^-1^, 5 weeks	↑	↑	↑	↓	Wu et al., 2016b
Asteraceae	*Eupatorium greggii*	10 dS·m^-1^, 5 weeks	↑	↑	↑	↓	Wu et al., 2016b
Asteraceae	*Gazania rigen*	12 dS·m^-1^, 12 weeks	↓	↑	↑		Niu and Rodriguez, 2006b
Asteraceae	*Gazania* sp*lendens*	7.5 dS·m^-1^, 60 days			↑	↑	García-Caparrós et al., 2016
Asteraceae	*Gerbera jamesonii*	~4.6 dS·m^-1^, 5 months			↑	↓	Don et al., 2010
Asteraceae	*Leucanthemum ×superbum*	10 dS·m^-1^, 5 weeks	↑	↑	↑	↓	Wu et al., 2016b
Asteraceae	*Melampodium leucanthum*	5–10 dS·m^-1^, 5 weeks	↑	↑	↑		Wu et al., 2016b
Asteraceae	*Osteospermum hybrida*	5 dS·m^-1^, 82 days		↑	↑	↓	Valdés et al., 2015
Asteraceae	*Rudbeckia fulgida*	16.1 dS·m^-1^, 6 weeks		↑	↑		Gerber et al., 2011
Asteraceae	*Santolina chamaecyparissus*	10 dS·m^-1^, 5 weeks	↑	↑	↑	↓	Wu et al., 2016b
Asteraceae	*Senecio cineraria*	13 dS·m^-1^, 30 days			↑	–	Saito et al., 2015
Asteraceae	*Symphyotrichum oblongifolium*	10 dS·m^-1^, 5 weeks	↑	↑	↑		Wu et al., 2016b
Asteraceae	*Tagetes lemmonii*	5–10 dS·m^-1^, 5 weeks	↑	↑	↑		Wu et al., 2016b
Asteraceae	*Tetraneuris scaposa*	10 dS·m^-1^, 5 weeks	↑	↑	↑	↓	Wu et al., 2016b
Asteraceae	*Viguiera stenoloba*	10 dS·m^-1^, 5 weeks	↑	↑	↑	↓	Wu et al., 2016b
Asteraceae	*Wedelia texana*	10 dS·m^-1^, 5 weeks	↑	↑	↑		Wu et al., 2016b
Asteraceae	*Zinnia maritima*	3-4.2 dS·m^-1^, 26 days	–	↑	↑	–	Niu et al., 2012c
Asteraceae	*Zinnia marylandica*	4.2 dS·m^-1^, 26 days	↑/–	↑	↑	↑/–	Niu et al., 2012c
Aizoaceae	*Delosperma cooperi*	12 dS·m^-1^, 12 weeks	–	↑	↑		Niu and Rodriguez, 2006b
Balsaminaceae	*Impatiens walleriana*	3.1 dS·m^-1^	↑	↑	↑	↓	Kuehny and Morales, 1998
Begoniaceae	*Begonia semperflorens*	~3.6 dS·m^-1^, 12 weeks	↓		↑		Çiçek, 2023
Brassicaceae	*Nasturtium officinale*	9.7 dS·m^-1^, 21 days		↑	↑	↓	Kaddour et al., 2013
Caryophyllaceae	*Dianthus chinensis*	7.8 dS·m^-1^, 39 days	↓		↑	↓	Zhang et al., 2019
Cannaceae	*Canna indica*	5–20 dS·m^-1^, 20 days			↑	↑	Chen et al., 2019b
Campanulaceae	*Lobelia cardinalis*	10 dS·m^-1^, 8 weeks	↑	↑	↑	↓	Wu et al., 2016a
Campanulaceae	*Lobelia erinus*	2 dS·m^-1^, 60 days	↓	↑	↑	↓	Escalona et al., 2013
Caprifoliacea	*Diervilla rivularis*	10 dS·m^-1^, 8 weeks	↑	↑	↑	↑	Liu et al., 2017
Caprifoliaceae	*Lonicera japonica*	5.4 dS·m^-1^, 103 days					Niu et al., 2007
Caprifoliaceae	*Scabiosa columbaria*	10 dS·m^-1^, 8 weeks	↑	↑	↑	↓	Wu et al., 2016a
Cleomaceae	*Cleome gynandra*	~6.9 dS·m^-1^, 5 weeks					Mwai et al., 2002
Convolvulaceae	*Evolvulus glomeratus*	5–10 dS·m^-1^, 8 weeks	↓	↑	↑	↓	Hooks and Niu, 2019
Convolvulaceae	*Ipomoea purpurea*	~12.9 dS·m^-1^, 3 weeks	–	↑	–	–	Mircea et al., 2023
Convolvulaceae	*Ipomoea tricolor*	~12.9 dS·m^-1^, 3 weeks	↑	↑	↑	↑	Mircea et al., 2023
Cornaceae	*Cornus alba*	10 dS·m^-1^, 8 weeks	↑	↑	↑	↑	Liu et al., 2020
Crassulaceae	*Sedum telephium*	10 dS·m^-1^, 8 weeks	↓	↑	↑	↓	Hooks and Niu, 2019
Crassulaceae	*Sedum reflexum*	10 dS·m^-1^, 8 weeks	–	↑	↑	↓	Hooks and Niu, 2019
Crassulaceae	*Sedum rupestre*	10 dS·m^-1^, 8 weeks	↓	↑	↑	↓	Hooks and Niu, 2019
Elaeagnaceae	*Shepherdia ×utahensis*	10 dS·m^-1^, 8 weeks	↑	↑	↑	↓	Paudel and Sun, 2023
Ericaceae	*Arctostaphylos uva-ursi*	10 dS·m^-1^, 8 weeks	↑	↑	↑	–	Paudel and Sun, 2023
Euphorbiaceae	*Euphorbia lathyris*	10.3-43.5 dS·m^-1^, 20 days	–		↑	↑, then ↓	Yang et al., 2013
Euphorbiaceae	*Jatropha curcas*	9 dS·m^-1^, 54 days		↑	↑		Niu et al., 2012b
Fabaceae	*Albizia julibrissin*	10 dS·m^-1^, 8 weeks	↑	↑	↑	↑	Paudel and Sun, 2022
Fabaceae	*Cercis canadensis*	3–6 dS·m^-1^, 167 days		↑	↑		Niu et al., 2010a
Fabaceae	*Sophora japonica*	5–10 dS·m^-1^, 8 weeks	↑	↑	–	–	Paudel and Sun, 2022
Fabaceae	*Sophora secundiflora*	3–6 dS·m^-1^, 194 days		↑	↑		Niu et al., 2010a
Gentianaceae	*Lisianthus* spp.	12 dS·m^-1^, until flowering		↑	↑		Valdez-Aguilar et al., 2013
Gentianaceae	*Lisianthus* spp.	12 dS·m^-1^, until flowering		↑	↑		Valdez-Aguilar et al., 2014
Gentianaceae	*Lisianthus* spp.	8.5 dS·m^-1^, 70 days	↓	↑	↑	↓	Ashrafi and Rezaei Nejad, 2018
Geraniaceae	*Pelargonium ×hortorum*	6.5 dS·m^-1^, 88 days		↑	↑		Valdés et al., 2015
Geraniaceae	*Pelargonium graveolens*	8.5 dS·m^-1^, 30 days	↑		↑	↓	Chrysargyris et al., 2021
Hydrangeaceae	*Dichroa febrifuga ×Hydrangea macrophylla*	10 dS·m^-1^, 52 days	↑	↑	↑	↓/–	Sun et al., 2022
Hydrangeaceae	*Hydrangea macrophylla*	10 dS·m^-1^, 4 weeks	↑	↑	↑	↑/↓/–	Niu et al., 2020
Hydrangeaceae	*Hydrangea macrophylla*	10 dS·m^-1^, 8 weeks	↑	↑	↑	–	Liu et al., 2017
Hydrangeaceae	*Hydrangea paniculata*	10 dS·m^-1^, 4 weeks	↑/–	↑	↑/–	↑/–	Niu et al., 2020
Hydrangeaceae	*Hydrangea quercifolia*	10 dS·m^-1^, 4 weeks	↑	↑	↑	↑	Niu et al., 2020
Hydrangeaceae	*Hydrangea serrata*	10 dS·m^-1^, 4 weeks	↑	↑	↑	↑	Niu et al., 2020
Hydrangeaceae	*Hydrangea serrata ×macrophylla*	10 dS·m^-1^, 4 weeks	↑	↑	↑	↓	Niu et al., 2020
Juncaceae	*Juncus effusus*	5–10 dS·m^-1^, 8 weeks					Sun and Palmer, 2018
Lamiaceae	*Agastache cana*	4 dS·m^-1^, 10 weeks					Niu and Rodriguez, 2006a
Lamiaceae	*Ajuga reptans*	10 dS·m^-1^, 6 weeks	↑	↑	↑	↓	Wu et al., 2016c
Lamiaceae	*Caryopteris ×clandonensis*	10 dS·m^-1^, 8 weeks	↓	↑	↑	↑	Wu et al., 2016a
Lamiaceae	*Lamium maculatum*	10 dS·m^-1^, 6 weeks	↑	↑	↑	–	Wu et al., 2016b
Lamiaceae	*Perovskia atriplicifolia*	10 dS·m^-1^, 6 weeks	↑	↑	↑	↓	Wu et al., 2016c
Lamiaceae	*Poliomintha longiflora*	10 dS·m^-1^, 6 weeks	↑	↑	↑	–	Wu et al., 2016c
Lamiaceae	*Salvia farinacea*	10 dS·m^-1^, 8 weeks	↑	↑	↑	–	Sun et al., 2015a
Lamiaceae	*Salvia leucantha*	5–10 dS·m^-1^, 8 weeks	↑	↑	↑	–	Sun et al., 2015a
Lamiaceae	*Scutellaria suffrutescens*	10 dS·m^-1^, 6 weeks	↑	↑	↑	–	Wu et al., 2016c
Lamiaceae	*Stachys coccinea*	10 dS·m^-1^, 6 weeks	↑	↑	↑	↓	Wu et al., 2016c
Lamiaceae	*Teucrium chamaedrys*	12 dS·m^-1^, 12 weeks	–	↑	↑		Niu and Rodriguez, 2006b
Lythraceae	*Cuphea hyssopifolia*	10 dS·m^-1^, 8 weeks	↑	↑	↑	↑	Wu et al., 2016a
Malvaceae	*Hibiscus syriacus*	10 dS·m^-1^, 8 weeks	↑	↑	↑	↑	Liu et al., 2017
Malvaceae	*Malvaviscus arboreus*	10 dS·m^-1^, 8 weeks	↑	↑	↑	↓	Sun et al., 2015a
Malvaceae	*Pavonia lasiopetala*	10 dS·m^-1^, 8 weeks	↑	↑	↑	–	Wu et al., 2016a
Oleaceae	*Forsythia ×intermedia*	10 dS·m^-1^, 8 weeks	↑	↑	↑	↑	Liu et al., 2017
Papaveracea	*Glaucium flavum*	~38.6 dS·m^-1^, 60 days	↓		↑	↑	Cambrollé et al., 2011
Plantaginaceae	*Angelonia angustifolia*	7.4 dS·m^-1^, 122 days		↑	↑		Niu et al., 2010a
Plantaginaceae	*Antirrhinum majus*	~5.2 dS·m^-1^, 76 days		↑	↑	↓	El-Attar, 2017
Plantaginaceae	*Antirrhinum majus*	14 dS·m^-1^, 42 days	↑	↑	↑	↓	Carter and Grieve, 2008
Plantaginaceae	*Bacopa monneiri*	~7.4 dS·m^-1^, 20 days		↑	↑		Khaliel et al., 2011
Plantaginaceae	*Penstemon barbatus*	10 dS·m^-1^, 8 weeks	↑	↑	↑	–	Paudel and Sun, 2024
Plantaginaceae	*Penstemon davidsonii*	2.5–10 dS·m^-1^, 8 weeks	↑	↑	↑	↑	Nepal et al., 2024
Plantaginaceae	*Penstemon eatonii*	12 dS·m^-1^, 10 weeks					Niu and Rodriguez, 2006b
Plantaginaceae	*Penstemon heterophyllus*	2.5–10 dS·m^-1^, 8 weeks	↑	↑	↑	–	Nepal et al., 2024
Plantaginaceae	*Penstemon strictus*	10 dS·m^-1^, 8 weeks	↑	↑	↑	↓	Paudel and Sun, 2024
Plumbaginaceae	*Ceratostigma plumbaginoides*	6.4 dS·m^-1^, 12 weeks	–	↑	↑		Niu and Rodriguez, 2006b
Poaceae	*Eragrostis* sp*ectabilis*	10 dS·m^-1^, 65 days	↑	↑	↑	↓	Wang et al., 2019b
Poaceae	*Miscanthus sinensis*	10 dS·m^-1^, 65 days	↑	↑	↑	–	Wang et al., 2019b
Poaceae	*Panicum virgatum*	10 dS·m^-1^, 65 days	↑	↑	↑	↓	Wang et al., 2019b
Poaceae	*Panicum virgatum*	10 dS·m^-1^, 4 weeks	↑/–	–	–/↑	–/↓	Sun et al., 2018a
Poaceae	*Pennisetum americanum*	20 dS·m^-1^, 4 weeks	↑	↑	↑	↑	Ashraf and McNeilly, 1987
Poaceae	*Schizachyrium scoparium*	10 dS·m^-1^, 65 days	↑	↑	↑	↓	Wang et al., 2019b
Poaceae	*Zoysia matrella*	10 dS·m^-1^, 8 weeks		↑	↑		Hooks et al., 2022
Poaceae	*Zoysia minima*	10 dS·m^-1^, 8 weeks		↑	↑		Hooks et al., 2022
Poaceae	*Zoysia japonica*	10 dS·m^-1^, 8 weeks		↑	↑		Hooks et al., 2022
Polemoniaceae	*Phlox paniculata*	10 dS·m^-1^, 8 weeks	↑	↑	↑	↓/–	Sun et al., 2015a
Ranunculaceae	*Aquilegia canadensis*	10 dS·m^-1^, 8 weeks	↑	↑	↑	–	Wu et al., 2016c
Ranunculaceae	*Ranunculus asiaticus*	6 dS·m^-1^, 88 days	↓			↓	Valdez-Aguilar et al., 2009
Ranunculaceae	*Ranunculus acris*	5.8 dS·m^-1^, 48 days	–		–	–	Wala et al., 2023
Ranunculaceae	*Ranunculus sceleratus*	15.6 dS·m^-1^, 5 weeks			↑	↑	Ievinsh et al., 2022
Rosaceae	*Cercocarpus ledifolius*	10 dS·m^-1^, 8 weeks	↑	↑	↑	–	Paudel and Sun, 2023
Rosaceae	*Cercocarpus montanus*	10 dS·m^-1^, 8 weeks	↑	↑	↑	–	Paudel and Sun, 2023
Rosaceae	*Chaenomeles* sp*eciosa*	10 dS·m^-1^, 8 weeks	↑	↑	↑	↑/–	Liu et al., 2017
Rosaceae	*Rosa fortuniana*	9 dS·m^-1^, 15 weeks		↑	–		Niu et al., 2008
Rosaceae	*Rosa ×hybrida*	8 dS·m^-1^, 54 days		↑	↑		Cai et al., 2014b
Rosaceae	*Rosa multiflora*	9 dS·m^-1^, 15 weeks		↑	↑		Niu et al., 2008
Rosaceae	*Rosa odorata*	9 dS·m^-1^, 15 weeks		↑	↑		Niu et al., 2008
Rosaceae	*Rosa* spp.	6.4 dS·m^-1^, 7 weeks	–	↑	↑	↓/–	Niu et al., 2013
Rosaceae	*Rosa* spp.	6.4 dS·m^-1^, 10 weeks	↓/–	↑	↑	↓/–	Niu et al., 2013
Rosaceae	*Rosa* spp.	10 dS·m^-1^, 43 days					Cai et al., 2014a
Solanaceae	*Capsicum annuum*	8.1 dS·m^-1^, 57 days	↑	↑	↑	↓	Niu et al., 2012a
Solanaceae	*Capsicum annuum*	4.1 dS·m^-1^, 74 days	–	↑	↑	↓	Niu et al., 2010b
Solanaceae	*Cestrum* spp.	10 dS·m^-1^, 8 weeks	↑	↑	↑	↓	Wu et al., 2016a
Solanaceae	*Nicotiana rustica*	6.4 dS·m^-1^, 100 days			↑	↓	Cusido et al., 1987
Solanaceae	*Petunia hybrid*	12.5 dS·m^-1^, 30 days	–	↑	↑	↓	Fornes et al., 2007
Verbenaceae	*Lantana camara*	5.1 dS·m^-1^, 175 days		↑	↑		Bañón et al., 2011
Verbenaceae	*Verbena ×hybrida*	10 dS·m^-1^, 8 weeks	↑	↑	↑	↓	Sun et al., 2015a
Verbenaceae	*Verbena officinallis*	8.5 dS·m^-1^, 30 days	–		↑	↑	Chrysargyris et al., 2021
Violaceae	*Violax ×Wittrockiana*	3.1 dS·m^-1^, 8 weeks	↑	↑	↑	↓	Kuehny and Morales, 1998
Vitaceae	*Parthenocissus quinquefolia*	10 dS·m^-1^, 8 weeks	↑	↑	↑	–	Liu et al., 2017

i the electricity conductivity (EC) of saline irrigation.

^ii^ the duration of saline irrigation.

^iii^ no significant change observed on nutrition content.

^iv^ ↑, nutrition content significantly increased.

v ↓, nutrition content significantly decreased.

^vi^ no data collected.

Cl^-^ is an essential micronutrient for higher plants, playing a crucial role in various physiological processes (Broyer et al., 1954; Johnson et al., 1957). It functions as a major osmotically active solute and counter anion, contributing to the regulation of turgor pressure, intracellular pH gradients, and electrical excitability (White and Broadley, 2001). Moreover, Cl^-^ is involved in enzyme activation and is essential for photosynthesis (Geilfus, 2018; Raven, 2017). Cl^-^ deficiency leads to reduced leaf growth and wilting, followed by symptoms such as chlorosis, bronzing, and necrosis (White and Broadley, 2001). For example, Broyer et al. (1954) and Johnson et al. (1957) found that Cl^-^ deficiency caused severe growth inhibition and physiological stress, with *Lactuca sativa* (lettuce) and *Solanum lycopersicum* (tomato) being particularly sensitive. However, Cl^-^ deficiency is seldom observed under natural conditions, except in inland continental regions distant from the coast, where it becomes more pronounced in sandy soils (Geilfus, 2018; Yan et al., 2018). In non‐saline conditions, glycophytes actively uptake Cl^-^ and accumulate it in their leaves at concentrations comparable to those of other macronutrients, such as K^+^ and NO_3_^-^, thereby improving water relations, growth, and carbon (C), nitrogen (N), and energy metabolism, enhancing drought tolerance (Franco-Navarro et al., 2016; 2019, 2021; Peinado-Torrubia et al., 2023). In a saline context, similar to Na^+^, excessive Cl^-^ accumulation in plant tissues can lead to growth reduction and physiological stress (Cassaniti et al., 2013; Eaton, 1942; Geilfus, 2018). It is important to emphasize that Cl^-^ is the predominant anion in salinized soils (Geilfus, 2018). Cl^-^ toxicity symptoms typically begin with leaf discoloration, followed by necrotic lesions and leaf-tip burn, which can significantly impact the aesthetic value of ornamental plants (Cassaniti et al., 2009; García-Caparrós and Lao, 2018; Geilfus, 2018; Villarino and Mattson, 2011). As summarized in **Table 4**, most ornamental species exhibit significantly increased Cl^-^ content under saline conditions. However, *Panicum virgatum* appears to be an exception. Sun et al. (2018a) reported that after four weeks of saline irrigation at EC 10 dS·m^-1^, *P. virgatum* maintained Cl^-^ concentrations similar to that of control plants. This suggests that switchgrass possesses mechanisms to limit Cl^-^ accumulation in shoots, potentially contributing to its salinity tolerance.

As a key macronutrient, K^+^ is the most abundant cation in plant cells and the second most abundant nutrient in plant leaves after N (Prajapati and Modi, 2012; Sardans and Peñuelas, 2015). It plays a vital role in plant growth and metabolism, including enzyme activation, protein synthesis, stomatal regulation, ion absorption and transport, photosynthesis, respiration, and long-distance nutrient translocation (Mengel, 2016; Prajapati and Modi, 2012). Despite its importance, K^+^ availability in groundwater is generally low, as it is more easily leached than N and phosphorus (P), resulting in limited sources for plant uptake (Arienzo et al., 2009; Prajapati and Modi, 2012; Sardans and Peñuelas, 2015). K^+^ deficiency symptoms first appear in older leaves, manifesting as yellow scorching, chlorosis, and necrosis along the leaf margins (Prajapati and Modi, 2012; Mengel, 2016). Affected plants often exhibit slow growth and poorly developed root systems (Mengel, 2016). High salinity exacerbates nutrient imbalances, particularly by limiting K^+^ uptake due to competition with other monovalent cations, such as Na^+^ (Arif et al., 2020; Chérel et al., 2014). Salt-induced osmotic and ionic stresses impair K^+^ uptake efficiency, as Na^+^ competes for K^+^ binding sites, ultimately leading to chlorophyll degradation and protein dysfunction (Kumari et al., 2021). Under saline irrigation, 35.9% of ornamental species listed in **Table 4** experienced a significant decline in leaf K^+^ content (Cartmill et al., 2013; Don et al., 2010; Fornes et al., 2007; Kaddour et al., 2013; Sun et al., 2015a; Valdés et al., 2015; Zhang et al., 2019). Conversely, 17.9% of species exhibited an increase in K^+^ content under saline conditions, suggesting potential adaptive mechanisms for K^+^ retention or enhanced uptake (Ashraf and McNeilly, 1987; García-Caparrós et al., 2016; Ievinsh et al., 2022; Liu et al., 2017; Niu et al., 2020). Due to the simultaneous increase in Na^+^ and decrease in K^+^, most non-halophytic species exhibit a reduced K^+^/Na^+^ ratio under saline conditions. The ability to maintain K^+^ homeostasis and regulate the Na^+^/K^+^ ratio is crucial for salinity tolerance, as it helps mitigate the detrimental effects of salt stress and supports plant survival (Kumari et al., 2021; Sun et al., 2015b).

Ca²^+^ plays a vital role in maintaining ecosystem structure and function (Cramer, 2002; Luo et al., 2023). Although Ca²^+^ deficiency is generally rare as it is the fifth most abundant element in the Earth’s crust, soil Ca²^+^ can be lost due to water or wind erosion, leading to deficiency symptoms such as poor root development, leaf necrosis and curling, and blossom-end rot (Hepler, 2005; Luo et al., 2023; Thor, 2019; White and Broadley, 2003). As an essential macronutrient, Ca^2+^ serves both structural and signaling functions in plants. It plays a crucial role in cell wall and membrane stability, contributing to rigidity and overall plant integrity (Hepler, 2005; Thor, 2019). Plus, Ca^2+^ acts as a critical intracellular messenger, regulating numerous developmental and physiological processes (Thor, 2019; White and Broadley, 2003). A complex Ca^2+^ response network, consisting of Ca^2+^-integrated proteins, phytohormones, osmolytes, receptors, and other signaling factors, mediates cellular responses to abiotic stresses, including salinity (Bachani et al., 2022). Under saline stress, an increase in Ca^2+^ concentration often inhibits plant growth (Bressan et al., 1998; Porcelli et al., 1995). In roots, high extracellular NaCl triggers Ca^2+^ influx, elevating cytosolic Ca^2+^ levels as a secondary messenger in stress signaling pathways (Laohavisit et al., 2013). Several studies have demonstrated that supplementing Ca^2+^ effectively alleviates NaCl-induced stress, improving plant resilience to salinity (Nepal et al., 2024). As summarized in **Table 4**, 59.0% of ornamental plants exhibited a significant increase in Ca²^+^ content when irrigated with saline water, suggesting a compensatory mechanism in response to saline stress. In contrast, only 10.2% of species showed a significant decline in Ca²^+^ content, highlighting the generally protective role of Ca²^+^ in maintaining plant function under saline conditions.

### 3.4 Photosynthetic characteristics

#### 3.4.1 Chlorophyll

Chlorophyll is crucial component of ornamental plants as it enhances leaf greenness, which improves their aesthetic appeal, and plays a pivotal role in converting sunlight into chemical energy through photosynthesis (Xiong et al., 2015). Both biotic and abiotic stresses have impacts on content and efficiency of leaf photosynthetic pigments, including chlorophylls, carotenoids, and anthocyanins, which often lead to changes specifically in chlorophyll content (Percival et al., 2008). Environmental stress, for instance, can inhibit chlorophyll synthesis while also triggering its degradation (Taïbi et al., 2016). As a result, reduced chlorophyll content became a typical indicator of oxidative stress and leaf senescence in plants (Kräutler, 2009; Taïbi et al., 2016). Consequently, “leaf greenness” serves as a robust indicator of plant vitality and stress levels (Percival et al., 2008).

**Table 5** summarizes the leaf greenness responses of 72 ornamental species across 28 botanical families under varying levels of saline irrigation. *Cleome gynand*ra exhibited an 80% reduction in chlorophyll content when exposed to saline irrigation at EC 6.9 dS·m^-1^ for 5 weeks (Mwai et al., 2002). In contrast, species like *Begonia semperflorens*, *Calendula officinalis*, and *Catharanthus roseus* experienced decreases of less than 50% in chlorophyll content when irrigated with saline solutions at EC ranging from 3.6 to 12.5 dS·m^-1^ (Çiçek, 2023; Fornes et al., 2007; Mohammadi Kabari et al., 2024). Some species, such as *Ageratum conyzoides*, *Ipomoea tricolor*, and *Ranunculus acris*, maintained similar chlorophyll content under saline stress, with reductions of less than 6% after irrigation periods of 20 to 48 days at EC between 5.8 to 12.9 dS·m^-1^ (Mircea et al., 2023; Sun et al., 2012; Wala et al., 2023). While saline stress typically reduces chlorophyll content, certain studies have reported increased levels under such conditions (Gómez et al., 2003; Shah et al., 2017). For example, *Petunia hybrid* exhibited enhanced chlorophyll content when subjected to saline irrigation at EC 12.5 dS·m^-1^ for 30 days, likely because it accumulated more N and Mg^2+^, which are essential components of chlorophyll molecules, demonstrating high salt tolerance (Fornes et al., 2007).

**Table 5:** Effects of leaf greenness parameters, including chlorophyll content, soil plant analysis development (SPAD), and the maximum quantum efficiency of photosystem II (*F*_v_/*F*_m_) in different ornamental species.

Botanical family	Species	Salt treatments	Leaf greenness observation	References
Acanthaceae	*Anisacanthus quadrifidus*	5–10 dS·m^-1 i^, 8 weeks^ii^	SPAD reduction of 11%-17%	Wu et al., 2016a
Acanthaceae	*Dicliptera suberecta*	5–10 dS·m^-1^, 8 weeks	SPAD reduction of 5%	Wu et al., 2016a
Acanthaceae	*Ruellia brittoniana*	5–10 dS·m^-1^, 8 weeks	SPAD reduction of 4%	Sun et al., 2015a
Amaranthaceae	*Celosia argentea*	~7.7 dS·m^-1^	Significant reduction on chlorophyll a content	Gholamzadeh Alam et al., 2022
Apocynaceae	*Catharanthus roseus*	4.7 dS·m^-1^, 4 months	Chlorophyll content reduction on 40%	Mohammadi Kabari et al., 2024
Asteraceae	*Ageratum conyzoides*	~1.6 dS·m^-1^, 4 weeks	Chlorophyll content reduction of 30%	Putri et al., 2024
Asteraceae	*Ageratum conyzoides*	~1.6-9.7 dS·m^-1^, 20 days	Chlorophyll content not affected	Sun et al., 2012
Asteraceae	*Calendula officinalis*	~4.7 dS·m^-1^, 137 days	SPAD reduction of 36%	Swaefy and El-Ziat, 2020
Asteraceae	*Calendula officinalis*	9.7 dS·m^-1^, 4 weeks	Chlorophyll a and b content reductions of 50% and 40%, respectively	Kozminska et al., 2017
Asteraceae	*Calendula officinalis*	12.5 dS·m^-1^, 70 days	Chlorophyll content reduction of 24%	Fornes et al., 2007
Asteraceae	*Gaillardia aristata*	5.4 dS·m^-1^, 103 days	SPAD not affected	Niu et al., 2007
Asteraceae	*Gerbera jamesonii*	~12.9 dS·m^-1^, 10 hours	Significant reduction on chlorophyll content	Uzma et al., 2022b
Asteraceae	*Gerbera jamesonii*	~12.9 dS·m^-1^, 20 days	Significant reduction on chlorophyll content	Uzma et al., 2022a
Asteraceae	*Melampodium leucanthum*	10 dS·m^-1^, 5 weeks	SPAD reduction of 28%	Wu et al., 2016b
Asteraceae	*Osteospermum hybrida*	5 dS·m^-1^, 82 days	SPAD not affected	Valdés et al., 2015
Asteraceae	*Tagetes erecta*	3–6 dS·m^-1^, 8 weeks	SPAD reduction of 19%-61%	Sun et al., 2018b
Asteraceae	*Tagetes lemmonii*	5–10 dS·m^-1^, 5 weeks	SPAD reduction of 46%	Wu et al., 2016b
Asteraceae	*Wedelia texana*	10 dS·m^-1^, 5 weeks	SPAD reduction of 26%	Wu et al., 2016b
Balsaminaceae	*Impatiens walleriana*	3.9 dS·m^-1^	Chlorophyll reduction of 41%	Roozbahani et al., 2020
Begoniaceae	*Begonia semperflorens*	~3.6 dS·m^-1^, 12 weeks	Chlorophyll reduction of 38%	Çiçek, 2023
Brassicaceae	*Brassica oleracea*	~3.2-51.5 dS·m^-1^, 15 days	SPAD reduction of 58%	Salachna et al., 2017
Brassicaceae	*Nasturtium officinale*	2.8 dS·m^-1^, 19 days	Chlorophyll content not affected	Pavlova et al., 2021
Brassicaceae	*Nasturtium officinale*	9.7 dS·m^-1^, 21 days	Chlorophyll a and b content reductions of 64% and 48%, respectively	Kaddour et al., 2013
Cannaceae	*Canna indica*	5–10 dS·m^-1^, 20 days	Significant reduction on chlorophyll	Chen et al., 2019b
Campanulaceae	*Lobelia cardinalis*	5–10 dS·m^-1^, 8 weeks	SPAD reduction of 21%-25%	Wu et al., 2016a
Caprifoliacea	*Diervilla rivularis*	5 dS·m^-1^, 8 weeks	SPAD reduction of 24%-47%	Liu et al., 2017
Caprifoliaceae	*Lonicera japonica*	5.4 dS·m^-1^, 103 days	SPAD not affected	Niu et al., 2007
Caprifoliaceae	*Scabiosa columbaria*	5–10 dS·m^-1^, 8 weeks	SPAD reduction of 2%-8%	Wu et al., 2016a
Cleomaceae	*Cleome gynandra*	~6.9 dS·m^-1^, 5 weeks	Chlorophyll reduction up to 80%	Mwai et al., 2002
Convolvulaceae	*Ipomoea purpurea*	~12.9 dS·m^-1^, 3 weeks	Significant reduction on chlorophyll a content	Mircea et al., 2023
Convolvulaceae	*Ipomoea tricolor*	~12.9 dS·m^-1^, 3 weeks	Chlorophyll a content not affected	Mircea et al., 2023
Cyperaceae	*Carex morrowii*	10 dS·m^-1^, 95 days	SPAD reduction of 36%	Xing et al., 2021
Gentianaceae	*Lisianthus* spp.	8.5 dS·m^-1^, 70 days	Chlorophyll reduction of 18%	Ashrafi and Rezaei Nejad, 2018
Geraniaceae	*Pelargonium ×hortorum*	6.5 dS·m^-1^, 88 days	SPAD reduction of 13%	Valdés et al., 2015
Geraniaceae	*Pelargonium graveolens*	8.5 dS·m^-1^, 30 days	*F*_v_/*F*_m_ and SPAD reductions of 5% and 24%, respectively	Chrysargyris et al., 2021
Hydrangeaceae	*Dichroa febrifuga ×Hydrangea macrophylla*	10 dS·m^-1^, 52 days	Significantly reduced on SPAD	Sun et al., 2022
Hydrangeaceae	*Hydrangea macrophylla*	5–10 dS·m^-1^, 8 weeks	SPAD reduction of 10%-21%	Liu et al., 2017
Lamiaceae	*Caryopteris ×clandonensis*	5–10 dS·m^-1^, 8 weeks	SPAD reduction of 26%	Wu et al., 2016a
Lamiaceae	*Lamium maculatum*	5–10 dS·m^-1^, 6 weeks	SPAD reduction of 14%	Wu et al., 2016c
Lamiaceae	*Salvia farinacea*	10 dS·m^-1^, 8 weeks	SPAD reduction of 12%	Sun et al., 2015a
Lamiaceae	*Salvia leucantha*	5–10 dS·m^-1^, 8 weeks	SPAD reduction of 6%-23%	Sun et al., 2015a
Lamiaceae	*Scutellaria suffrutescens*	5 dS·m^-1^, 6 weeks	SPAD reduction of 26%	Wu et al., 2016c
Malvaceae	*Hibiscus syriacus*	6.5 dS·m^-1^, 11 weeks	SPAD reduction of 35%	Chen et al., 2019a
Malvaceae	*Hibiscus syriacus*	5–10 dS·m^-1^, 8 weeks	SPAD reduction of 14%-73%	Liu et al., 2017
Malvaceae	*Malvaviscus arboreus*	5–10 dS·m^-1^, 8 weeks	SPAD reduction of 4%-6%	Sun et al., 2015a
Malvaceae	*Pavonia lasiopetala*	5–10 dS·m^-1^, 8 weeks	SPAD reduction of 1%-8%	Wu et al., 2016a
Oleaceae	*Forsythia ×intermedia*	5–10 dS·m^-1^, 8 weeks	SPAD reduction of 2%-17%	Liu et al., 2017
Papaveracea	*Glaucium flavum*	~19.3 dS·m^-1^, 60 days	Chlorophyll reduction of 50%	Cambrollé et al., 2011
Plantaginaceae	*Antirrhinum majus*	~5.2 dS·m^-1^, 76 days	SPAD reduction of 10%	El-Attar, 2017
Plantaginaceae	*Bacopa monneiri*	~7.4 dS·m^-1^, 20 days	Chlorophyll significantly reduced	Khaliel et al., 2011
Plantaginaceae	*Penstemon barbatus*	5-7.5 dS·m^-1^, 8 weeks	SPAD reduction of 28%-71%	Paudel and Sun, 2024
Plantaginaceae	*Penstemon heterophyllus*	5-7.5 dS·m^-1^, 8 weeks	SPAD reduction of 26%-40%	Nepal et al., 2024
Poaceae	*Bouteloua gracilis*	5–10 dS·m^-1^, 18 weeks	SPAD not affected	Sun and Palmer, 2018
Poaceae	*Eragrostis* sp*ectabilis*	10 dS·m^-1^, 65 days	SPAD reduction of 2%	Wang et al., 2019b
Poaceae	*Leymus arenarius*	5–10 dS·m^-1^, 18 weeks	SPAD not affected	Sun and Palmer, 2018
Poaceae	*Miscanthus sinensis*	10 dS·m^-1^, 65 days	SPAD reduction of 3%-10%	Wang et al., 2019b
Poaceae	*Muhlenbergia capillaris*	10 dS·m^-1^, 18 weeks	SPAD not affected	Sun and Palmer, 2018
Poaceae	*Panicum virgatum*	10 dS·m^-1^, 65 days	SPAD reduction of 6%-9%	Wang et al., 2019b
Poaceae	*Panicum virgatum*	10 dS·m^-1^, 4 weeks	SPAD reduction of 17%	Sun et al., 2018a
Poaceae	*Schizachyrium scoparium*	10 dS·m^-1^, 65 days	SPAD reductions of 3%-5%	Wang et al., 2019b
Poaceae	*Sporobolus heterolepis*	5–10 dS·m^-1^, 95 days	SPAD reductions of 20%	Xing et al., 2021
Ranunculaceae	*Aquilegia canadensis*	5–10 dS·m^-1^, 8 weeks	SPAD reduction of 16%-25%	Wu et al., 2016c
Ranunculaceae	*Ranunculus acris*	5.8 dS·m^-1^, 48 days	Chlorophyll reduction of 6%	Wala et al., 2023
Rosaceae	*Chaenomeles* sp*eciosa*	5 dS·m^-1^, 8 weeks	SPAD reduction of 32%-33%	Liu et al., 2017
Rosaceae	*Physocarpus opulifolius*	4.6-6.5 dS·m^-1^, 11 weeks	SPAD reduction of 21%	Chen et al., 2019a
Rosaceae	*Spiraea japonica*	3–6 dS·m^-1^, 8 weeks	SPAD reduction of 11%-27%	Wang et al., 2019a
Rosaceae	*Spiraea japonica*	6.5 dS·m^-1^, 11 weeks	SPAD reduction of 35%	Chen et al., 2019a
Rosaceae	*Rosa ×hybrida*	8 dS·m^-1^, 54 days	*F*_v_/*F*_m S_ignificantly reduced	Cai et al., 2014b
Rosaceae	*Rosa* spp.	10 dS·m^-1^, 43 days	SPAD reduction of 11%-28%	Cai et al., 2014a
Solanaceae	*Capsicum annuum*	4.1 dS·m^-1^, 74 days	SPAD not affected	Niu et al., 2010c
Solanaceae	*Cestrum* spp.	10 dS·m^-1^, 8 weeks	SPAD reductions of 7%	Wu et al., 2016a
Solanaceae	*Petunia hybrid*	12.5 dS·m^-1^, 30 days	Chlorophyll content increase of 12%	Fornes et al., 2007
Verbenaceae	*Glandularia canadensis*	3.2-5.4 dS·m^-1^, 103 days	SPAD reductions of 7%-14%	Niu et al., 2007
Verbenaceae	*Glandularia ×hybrida*	3.2-5.4 dS·m^-1^, 103 days	SPAD not affected	Niu et al., 2007
Verbenaceae	*Lantana montevidensis*	3.2-5.4 dS·m^-1^, 103 days	SPAD reductions of 35%-47%	Niu et al., 2007
Verbenaceae	*Lantana ×hybrida*	5.4 dS·m^-1^, 103 days	SPAD not affected	Niu et al., 2007
Verbenaceae	*Verbena ×hybrida*	5–10 dS·m^-1^, 8 weeks	SPAD reduction of 2%-10%	Sun et al., 2015a
Verbenaceae	*Verbena macdougalii*	5.4 dS·m^-1^, 103 days	SPAD not affected	Niu et al., 2007
Verbenaceae	*Verbena officinallis*	3.9 dS·m^-1^, 12 weeks	Chlorophyll reduction of 34%	Çiçek and Yücedağ, 2023
Verbenaceae	*Verbena officinallis*	8.5 dS·m^-1^, 30 days	*F*_v_/*F*_m_ and SPAD reductions of 7% and 33%, respectively	Chrysargyris et al., 2021
Vitaceae	*Parthenocissus quinquefolia*	5–10 dS·m^-1^, 8 weeks	SPAD reduction of 8%-15%	Liu et al., 2017

i the electricity conductivity (EC) of saline irrigation.

^ii^the duration of saline irrigation.

Measuring chlorophyll content *in vitro* is considered more accurate but involves complex, destructive, and resource-intensive procedures (Nagaoka, 2022; Shah et al., 2017; Uddling et al., 2007). In contrast, non-destructive methods such as handheld meters, such as the SPAD-502 meter, are gaining popularity. This SPAD-502 device calculates SPAD readings based on the differential absorption of red (650 nm) and infrared (940 nm) light by chlorophyll in plant tissue (Percival et al., 2008; Xiong et al., 2015). **Table 5** also highlights species that maintained unaffected SPAD readings under saline irrigation, including *Capsicum annuum*, *Gaillardia aristata*, and *Muhlenbergia capillaris* (Niu et al., 2007, 2010c; Sun and Palmer, 2018). Conversely, species like *Antirrhinum majus*, *Anisacanthus quadrifidus*, *Pelargonium ×hortorum*, and *Ruellia brittoniana* experienced a less than 20% reduction in SPAD readings after exposure to saline solutions ranging from 5 to 10 dS·m^-1^ for periods of 8 weeks to 88 days (El-Attar, 2017; Sun et al., 2015a; Valdés et al., 2015; Wu et al., 2016a). However, some species showed significant declines in SPAD readings, up to 71% and 73%, as reported by Liu et al. (2017) and Paudel and Sun (2024), respectively, under similar saline treatments. The relationship between chlorophyll content and SPAD readings varies among species and is influenced by factors such as leaf orientation, sensor type, and measurement protocols (Xiong et al., 2015; Uddling et al., 2007). Furthermore, the interaction of SPAD readings and abiotic stresses, including salinity, have received little attention (Shah et al., 2017).

#### 3.4.2 Quantum efficiency

The *F*_v_/*F*_m_ ratio provides an estimate of the maximum quantum efficiency of photosystem II (PSII) photochemistry. It also offers a rapid and accurate method to assess plant health and stress tolerance (Li et al., 2006; Lucena et al., 2012). For instance, *Pelargonium graveolens*, Rosa spp., and *Verbena officinallis* exhibited substantial declines in *F*_v_/*F*_m_ under saline irrigation (Cai et al., 2014b; Chrysargyris et al., 2021). PSII is considered the most heat-sensitive component of the photosynthetic apparatus (Čajánek et al., 1998; Zhou et al., 2015). Therefore, the significant fluctuation in canopy temperature, which is a consequence of stomatal closure under saline stress, can lead to reductions in *F*_v_/*F*_m_ (Acosta-Motos et al., 2017; Khasanov et al., 2023).

#### 3.4.3 Stomatal conductance

Stomata, which consists of pairs of guard cells, regulate gas exchange and water loss by adjusting the size of the stomatal pore through changes in turgor and volume. This process is primarily controlled by K^+^ uptake, which is inhibited under salt stress (Hedrich and Shabala, 2018). As a result, stomatal conductance declines under saline conditions, making it a widely used parameter for screening osmotic stress tolerance (Jiang et al., 2006; Rahnama et al., 2010). The reduction of stomatal conductance varies across species and salinity levels. For instance, *Caryopteris ×clandonensis* and *Phlox paniculata* exhibited reductions ranging from 4-75% and 7-72%, respectively, when irrigated with saline water at EC between 5–10 dS·m^-1^ for 8 weeks (Sun et al., 2015a; Wu et al., 2016a). Similarly, some species showed minimal changes, such as *Anisacanthus quadrifidus* and *Ruellia brittoniana*, with reductions of less than 15% (Sun et al., 2015a; Wu et al., 2016a). Conversely, *Parthenocissus quinquefolia* exhibited a severe reduction of up to 92% under the same saline treatment (Liu et al., 2017). **Table 6** summarizes the stomatal and photosynthetic responses of 60 ornamental species from 30 botanical families under different levels of saline irrigation.

**Table 6:** Effects of saline irrigation on photosynthesis related parameters, including photosynthetic rate (*P*_n_), stomatal conductance (*g*_s_), and transpiration rate (*T*_r_) in different ornamental species.

Botanical family	Species	Salt treatments	Photosynthesis related observations	References
Acanthaceae	*Anisacanthus quadrifidus*	5–10 dS·m^-1 i^, 8 weeks^ii^	*P*_n_, *g*_s_, and *T*_r_ reductions of 25%-54%, 7%-15%, and 16%-31%, respectively	Wu et al., 2016a
Acanthaceae	*Dicliptera suberecta*	5–10 dS·m^-1^, 8 weeks	*P*_n_, *g*_s_, and *T*_r_ reductions of 10%-24%, 26%-33%, and 14%-22%, respectively	Wu et al., 2016a
Acanthaceae	*Ruellia brittoniana*	5–10 dS·m^-1^, 8 weeks	*P*_n_, *g*_s_, and *T*_r_ reductions of 2-20%, 4%-8%, and 15%-18%	Sun et al., 2015a
Acoraceae	*Acorus gramineus*	10 dS·m^-1^, 95 days	*P*_n_ reductions of 56%; *g*_s_ and *T*_r_ not affected	Xing et al., 2021
Adoxaceae	*Viburnum×burkwoodii*	5–10 dS·m^-1^, 8 weeks	*P*_n_ and *g*_s_ reductions of 74% and 60%, respectively, at EC10; significant reduction on *T*_r_ at EC5	Chen et al., 2020; Sun et al., 2020
Adoxaceae	*Viburnum×’NCVX1’*	10 dS·m^-1^, 8 weeks	*P*_n_ reduction of 85%	Chen et al., 2020; Sun et al., 2020
Adoxaceae	*Viburnum nudum*	5–10 dS·m^-1^, 8 weeks	*P*_n_ and *g*_s_ reductions of 91% and 88%, respectively	Chen et al., 2020; Sun et al., 2020
Adoxaceae	*Viburnum pragense*	5 dS·m^-1^, 8 weeks	*P*_n_ reduction of 59%	Chen et al., 2020; Sun et al., 2020
Adoxaceae	*Viburnum×rhytidophylloides*	5 dS·m^-1^, 8 weeks	*P*_n_ reduction of 61%	Chen et al., 2020; Sun et al., 2020
Amaranthaceae	*Celosia argentea*	~7.7 dS·m^-1^	Significant reduction on *P*_n_, *g*_s_, and *T*_r_	Gholamzadeh Alam et al., 2022
Asteraceae	*Ageratum conyzoides*	~7.9 dS·m^-1^, 4 weeks	Stomatal density increased	Putri et al., 2024
Brassicaceae	*Brassica oleracea*	~3.2-51.5 dS·m^-1^, 15 days	*g*_s_ reduction of 34%	Salachna et al., 2017
Brassicaceae	*Brassica* spp.	~12.9 dS·m^-1^, 24 hours	*P*_n_ reduction of 42%-67%	Pavlović et al., 2019
Campanulaceae	*Lobelia cardinalis*	5–10 dS·m^-1^, 8 weeks	*P*_n_, *g*_s_, and *T*_r_ reductions of 18%-54%, 6%-39%, and 13%-30%, respectively	Wu et al., 2016a
Caprifoliacea	*Diervilla rivularis*	5 dS·m^-1^, 8 weeks	*P*_n_, *g*_s_, and *T*_r_ reductions of 75%-91%, 58%-77%, and 43%-67%, respectively	Liu et al., 2017
Caprifoliaceae	*Scabiosa columbaria*	5–10 dS·m^-1^, 8 weeks	*P*_n_, *g*_s_, and *T*_r_ reductions of 5%-30%, 27%-55%, 14%-27%, respectively	Wu et al., 2016a
Cyperaceae	*Carex morrowii*	10 dS·m^-1^, 95 days	*P*_n_, *g*_s_, and *T*_r_ reductions of 69%, 50%, and 43%, respectively	Xing et al., 2021
Elaeagnaceae	*Shepherdia ×utahensis*	10 dS·m^-1^, 8 weeks	*P*_n_ and *g*_s_ reductions of 52% and 85%, respectively; *T*_r_ not affected	Paudel and Sun, 2023
Ericaceae	*Arctostaphylos uva-ursi*	5 dS·m^-1^, 8 weeks	*P*_n_ reduction of 52%; significant reduction on *T*_r_	Paudel and Sun, 2023
Euphorbiaceae	*Euphorbia milii*	5 dS·m^-1^, 50 days	*P*_n_ reduction of 74%	Santos et al., 2022
Fabaceae	*Albizia julibrissin*	5–10 dS·m^-1^, 8 weeks	*P*_n_, *g*_s_, and *T*_r_ reductions of 44%-72%, 53%-73%, and 48%-70%, respectively	Paudel and Sun, 2022
Fabaceae	*Sophora japonica*	5–10 dS·m^-1^, 8 weeks	*P*_n_, *g*_s_, and *T*_r_ reductions of 49%-66%, 75%, and 71%, respectively	Paudel and Sun, 2022
Fabaceae	*Sophora secundiflora*	3–6 dS·m^-1^, 194 days	*P*_n_, *g*_s_, and *T*_r_ reductions of 30%, 38%, and 24%, respectively	Niu et al., 2010a
Gentianaceae	*Lisianthus* spp.	8.5 dS·m^-1^, 70 days	*P*n, *g*s, and *T*_r_ reductions of 41%, 30%, and 34%, respectively	Ashrafi and Rezaei Nejad, 2018
Geraniaceae	*Pelargonium ×hortorum*	6.5 dS·m^-1^, 88 days	*P*_n_ and *g*_s_ reductions of 81% and 52%, respectively	Valdés et al., 2015
Goodeniaceae	*Scaevola sericea*	15.6 dS·m^-1^, 8 weeks	*P*_n_ and *g*_s_ reductions of 32% and 73%, respectively	Goldstein et al., 1996
Hydrangeaceae	*Dichroa febrifuga ×Hydrangea macrophylla*	5–10 dS·m^-1^, 52 days	*P*_n_, *g*_s_, and *T*_r_ reductions of 26%-63%, 32%-60%, and 26%-50%, respectively	Sun et al., 2022
Hydrangeaceae	*Hydrangea macrophylla*	5–10 dS·m^-1^, 8 weeks	*P*_n_, *g*_s_, and *T*_r_ reductions of 80%-210%, 26%-77%, 37%-61%, respectively	Liu et al., 2017
Lamiaceae	*Ajuga reptans*	5 dS·m^-1^, 6 weeks	*P*_n_, *g*_s_, and *T*_r_ reduction of 19%, 32%, and 16%, respectively	Wu et al., 2016c
Lamiaceae	*Caryopteris ×clandonensis*	5–10 dS·m^-1^, 8 weeks	*P*_n_, *g*_s_, and *T*_r_ reduction of 16%-60%, 4%-75%, and 1%-54%, respectively	Wu et al., 2016a
Lamiaceae	*Lamium maculatum*	5–10 dS·m^-1^, 6 weeks	*P*_n_, *g*_s_, and *T*_r_ reduction of 29%-37%, 47%-48%, and 25%, respectively	Wu et al., 2016c
Lamiaceae	*Perovskia atriplicifolia*	10 dS·m^-1^, 6 weeks	*g*_s_ and *T*_r_ reductions of 34% and 23%, respectively	Wu et al., 2016c
Lamiaceae	*Salvia farinacea*	5–10 dS·m^-1^, 8 weeks	*P*_n_, *g*_s_, and *T*_r_ reduction of 6%, 10%-19%, and 2%-10%, respectively	Sun et al., 2015a
Lamiaceae	*Salvia leucantha*	5–10 dS·m^-1^, 8 weeks	*P*_n_, *g*_s_, and *T*_r_ reduction of 26%-53%, 29%-42%, and 4%-31%, respectively	Sun et al., 2015a
Malvaceae	*Hibiscus syriacus*	6.5 dS·m^-1^, 11 weeks	*P*_n_ reduction of 52%	Chen et al., 2019a
Malvaceae	*Hibiscus syriacus*	5–10 dS·m^-1^, 8 weeks	*P*_n_, *g*_s_, and *T*_r_ reduction of 47%-97%, 53%-87%, and 31%-76%, respectively	Liu et al., 2017
Malvaceae	*Malvaviscus arboreus*	5–10 dS·m^-1^, 8 weeks	*P*_n_, *g*_s_, and *T*_r_ reduction of 6%-31%, 11%-45%, and 6%-40%, respectively	Sun et al., 2015a
Malvaceae	*Pavonia lasiopetala*	5–10 dS·m^-1^, 8 weeks	*P*_n_, *g*_s_, and *T*_r_ reduction of 5%-8%, 11%-34%, and 9%-23%, respectively	Wu et al., 2016a
Oleaceae	*Forsythia ×intermedia*	5–10 dS·m^-1^, 8 weeks	*P*_n_, *g*_s_, and *T*_r_ reduction of 28%-58%, 37%-53%, and 34%-43%, respectively	Liu et al., 2017
Papaveracea	*Glaucium flavum*	~38.6 dS·m^-1^, 60 days	Significant reduction on *P*_n_ and *g*_s_	Cambrollé et al., 2011
Plantaginaceae	*Penstemon barbatus*	2.5–5 dS·m^-1^, 8 weeks	*P*_n_, *g*_s_, and *T*_r_ reduction of 30%-59%, 65%, and 48%, respectively	Paudel and Sun, 2024
Plantaginaceae	*Penstemon heterophyllus*	5-7.5 dS·m^-1^, 8 weeks	*P*_n_, *g*_s_, and *T*_r_ reduction of 37%-53%, 78%, and 54%, respectively	Nepal et al., 2024
Poaceae	*Calamagrostis ×acutiflora*	5–10 dS·m^-1^, 95 days	*P*_n_, *g*_s_, and *T*_r_ not affected	Xing et al., 2021
Poaceae	*Eragrostis* sp*ectabilis*	5–10 dS·m^-1^, 65 days	*P*_n_, *g*_s_, and *T*_r_ reductions of 19%-48%, 34%-58%, and 20%-34%, respectively	Wang et al., 2019b
Poaceae	*Miscanthus sinensis*	10 dS·m^-1^, 65 days	*P*_n_, *g*_s_, and *T*_r_ reductions of 31%-36%, 41%-44%, and 20%-34%, respectively	Wang et al., 2019b
Poaceae	*Panicum virgatum*	10 dS·m^-1^, 65 days	*P*_n_, *g*_s_, and *T*_r_ reductions of 2%-35%, 6%-35%, and 20%-34%, respectively	Wang et al., 2019b
Poaceae	*Panicum virgatum*	10 dS·m^-1^, 4 weeks	*P*_n_, *g*_s_, and *T*_r_ not affetced	Sun et al., 2018a
Poaceae	*Schizachyrium scoparium*	10 dS·m^-1^, 65 days	*P*_n_, *g*_s_, and *T*_r_ reductions of 31%-59%, 37%-62%, and 20%-34%, respectively	Wang et al., 2019b
Poaceae	*Sporobolus heterolepis*	5–10 dS·m^-1^, 95 days	*P*_n_, *g*_s_, and *T*_r_ reductions of 83%, 57%, 53%, respectively	Xing et al., 2021
Polemoniaceae	*Phlox paniculata*	5–10 dS·m^-1^, 8 weeks	*P*_n_, *g*_s_, and *T*_r_ reductions of 6%-91%, 7%-72%, and 29%-76%, respectively	Sun et al., 2015a
Ranunculaceae	*Aquilegia canadensis*	5 dS·m^-1^, 8 weeks	*P*_n_, *g*_s_, and *T*_r_ reductions of 15%, 37%, and 24%, respectively	Wu et al., 2016c
Rosaceae	*Cercocarpus ledifolius*	5 dS·m^-1^, 8 weeks	*P*_n_ reduction of 32%; significant reduction on *T*_r_	Paudel and Sun, 2023
Rosaceae	*Cercocarpus montanus*	10 dS·m^-1^, 8 weeks	*P*_n_ reduction of 95%; significant reduction on *T*_r_	Paudel and Sun, 2023
Rosaceae	*Chaenomeles* sp*eciosa*	5 dS·m^-1^, 8 weeks	*P*_n_, *g*_s_, and *T*_r_ reductions of 37%-73%, 22%-61%, and 11%-47%, respectively	Liu et al., 2017
Rosaceae	*Physocarpus opulifolius*	4.6-6.5 dS·m^-1^, 11 weeks	*P*_n_ reduction of 21%	Chen et al., 2019a
Rosaceae	*Spiraea japonica*	3–6 dS·m^-1^, 8 weeks	*P*_n_ reduction of 41%-57% at EC10; *g*_s_ and *T*_r_ not affected at EC3; *g*_s_ and *T*_r_ reductions of 14% and 38%	Wang et al., 2019a
Rosaceae	*Spiraea japonica*	5.7 dS·m^-1^, 11 weeks	*P*_n_ reduction of 39%	Chen et al., 2019a
Rosaceae	*Rosa ×hybrida*	8 dS·m^-1^, 54 days	*g*_s_ reductions of 19%-36%	Cai et al., 2014b
Rosaceae	*Rosa* spp.	10 dS·m^-1^, 43 days	*P*_n_, *g*_s_, and *T*_r_ reductions of 19%-43%, 29%-49%, and 25%-26%, respectively	Cai et al., 2014a
Solanaceae	*Capsicum annuum*	4.1 dS·m^-1^, 74 days	*g*_s_ reduction of 46%	Niu et al., 2010c
Solanaceae	*Cestrum* spp.	10 dS·m^-1^, 8 weeks	*P*_n_, *g*_s_, and *T*_r_ reductions of 16%-27%, 28%, and 1%-7%, respectively	Wu et al., 2016a
Verbenaceae	*Verbena ×hybrida*	5–10 dS·m^-1^, 8 weeks	*P*_n_ and *g*_s_ reductions of 3%-23% and 5%-9%, respectively; *T*_r_ not affected	Sun et al., 2015a
Vitaceae	*Parthenocissus quinquefolia*	5–10 dS·m^-1^, 8 weeks	*P*_n_, *g*_s_, and *T*_r_ reductions of 12%-93%, 32%-92%, and 50%-75%, respectively	Liu et al., 2017

i the electricity conductivity (EC) of saline irrigation.

^ii^the duration of saline irrigation.

Transpiration is the primary process of water loss in plants, with stomatal transpiration accounting for approximately 95% of the total (Hedrich and Shabala, 2018; Sterling, 2005). A reduction in stomatal conductance under salt stress leads to a decline in transpiration rate, with responses varying across species and saline levels. For example, the transpiration rate of *Spiraea japonica* decreased by 38% under saline irrigation at EC 6 dS·m^-1^ but remained unchanged at EC 3 dS·m^-1^ over the same 8-week period (Wang et al., 2019a). *Acorus gramineus* showed no significant change in transpiration after 95 days of saline irrigation at EC 10 dS·m^-1^ (Xing et al., 2021). In contrast, *Albizia julibrissin* experienced a reduction of up to 73% when exposed to the same saline conditions for 8 weeks (Paudel and Sun, 2022).

#### 3.4.4 Photosynthesis

Photosynthesis is a fundamental process for plant growth and development but is highly sensitive to environmental stresses, including salinity (Li et al., 2006; Sudhir and Murthy, 2004; Zhang et al., 2011). Under salt stress, a decrease in leaf area can limit photosynthetic area, reducing growth and productivity (Paudel and Sun, 2022). Reduced transpiration rate can mitigate water loss under salt stress, however, it also limits CO_2_ diffusion into leaves, restricting photosynthetic efficiency and increasing leaf temperature due to reduced evaporative cooling (Farquhar and Sharkey, 1982; Zhu et al., 2022). At the early stages of salt stress, stomatal closure is the primary limitation to photosynthesis (Bose et al., 2017; Pan et al., 2021). Over time, progressive salt accumulation in plant tissues further inhibits CO_2_ assimilation by disrupting chloroplast function and reducing chlorophyll content, which is positively correlated with photosynthetic rate (Li et al., 2006; Sudhir and Murthy, 2004). In addition, salt stress disrupts enzymatic activity in photosynthetic process, such as the function of RuBPCO carboxylase (Rubisco) (Sudhir and Murthy, 2004; Zahra et al., 2022). The impact of salinity on photosynthesis varies among species. Some, like *Calamagrostis ×acutiflora* and *Panicum virgatum*, exhibited no significant reduction in photosynthesis when exposed to saline irrigation at EC between 5–10 dS·m^-1^ for 4 weeks to 95 days (Sun et al., 2020; Xing et al., 2021). Others, such as *Pavonia lasiopetala* and *Scabiosa columbaria*, showed relatively mild reductions (less than 30%) under similar conditions (Sun et al., 2015a; Wu et al., 2016a). However, more salt-sensitive species, including *Cercocarpus montanus*, *Diervilla rivularis*, *Hibiscus syriacus*, and *Viburnum nudum*, exhibited drastic reductions in photosynthetic rate (over 90%) after 8 weeks of exposure to saline irrigation at EC between 5–10 dS·m^-1^ (Chen et al., 2020; Liu et al., 2017; Paudel and Sun, 2023). These findings highlight the species-specific nature of photosynthetic responses to saline stress and the importance of selecting salt-tolerant ornamentals for saline environments.

## 4 Conclusion

For nurseries, the most critical factors under saline conditions include species-specific salt thresholds, exposure duration, and its impacts on aesthetics, growth, plant nutrition and physiology. Early signs such as chlorosis and reduced leaf expansion often suggest substantial declines in their marketability. Growers should regularly monitor irrigation water and soil EC, while selecting ornamental species with documented salt-tolerance to minimize production losses. Therefore, understanding species-specific responses to saline irrigation is essential for selecting suitable ornamentals for sustainable nursery production and landscape applications using low-quality water resources. In **Table 7**, some species such as *Ageratum conyzoides*, *Santolina chamaecyparissus*, *Zoysia matrella*, and *Z. japonica* maintained high visual quality and growth vigor at EC exceeding 9 dS·m^-1^, highlighting their application potential in arid and semi-arid regions where reclaimed or brackish water is commonly used. Meanwhile, other species like *Alyssum murale*, *Gazania rigens*, and *Glandularia canadensis* tolerated saline irrigation at EC ~3 dS·m^-1^, suggesting that they can be used with moderately low-quality water. These findings offer practical guidance for species selection and irrigation planning under salinity constraints.

**Table 7:** Saline irrigation thresholds of ornamental species ^i^.

Botanical family	Species	Saline threshold	Notes	References
Adoxaceae	*Viburnum* ×’NCVX1’	5 dS·m^-1^, 8 weeks ^ii^	High photosynthesis reduction indicates potential growth inhibition under prolonged saline irrigation	Chen et al., 2020; Sun et al., 2020
Asteraceae	*Achillea millefolium*	5.4 dS·m^-1^, 103 days		Niu et al., 2007
Asteraceae	*Ageratum conyzoides*	~9.7 dS·m^-1^, 20 days		Sun et al., 2012
Asteraceae	*Gazania rigen*	3.2 dS·m^-1^, 12 weeks		Niu and Rodriguez, 2006b
Asteraceae	*Santolina chamaecyparissus*	10 dS·m^-1^, 5 weeks		Wu et al., 2016b
Asteraceae	*Senecio cineraria*	13 dS·m^-1^, 30 days		Saito et al., 2015
Brassicaceae	*Alyssum murale*	~3.2 dS·m^-1^, 21 days		Comino et al., 2005
Caryophyllaceae	*Dianthus chinensis*	4.5 dS·m^-1^, 8 weeks	High growth reduction was observed when irrigation EC increased to 7.8 dS·m^-1^ for 39 days.	Devitt and Morris, 1987; Zhang et al., 2019
Cannaceae	*Canna indica*	5 dS·m^-1^, 20 days	Substantial chlorophyll loss may precede visible damage and suggest future declines in plant vitality under continued salt exposure	Chen et al., 2019b
Caprifoliaceae	*Lonicera japonica*	5.4 dS·m^-1^, 103 days		Niu et al., 2007
Convolvulaceae	*Ipomoea tricolor*	~12.9 dS·m^-1^, 3 weeks		Mircea et al., 2023
Euphorbiaceae	*Euphorbia lathyris*	18.7 dS·m^-1^, 20 days		Yang et al., 2013
Fabaceae	*Sophora secundiflora*	3 dS·m^-1^, 194 days		Niu et al., 2010a
Geraniaceae	*Pelargonium graveolens*	8.5 dS·m^-1^, 30 days		Chrysargyris et al., 2021
Lamiaceae	*Caryopteris ×clandonensis*	5 dS·m^-1^, 8 weeks		Wu et al., 2016a
Lamiaceae	*Rosmarinus officinalis*	5.4 dS·m^-1^, 103 days		Niu et al., 2007
Lamiaceae	*Salvia farinacea*	5 dS·m^-1^, 8 weeks		Sun et al., 2015a
Lythraceae	*Cuphea hyssopifolia*	5 dS·m^-1^, 8 weeks		Wu et al., 2016a
Malvaceae	*Hibiscus syriacus*	6.5 dS·m^-1^, 11 weeks	High growth reduction was observed when irrigation EC increased to 10 dS·m^-1^ for 8 days.	Chen et al., 2019a; Liu et al., 2017
Plantaginaceae	*Penstemon davidsonii*	5 dS·m^-1^, 8 weeks		Nepal et al., 2024
Poaceae	*Bouteloua gracilis*	5 dS·m^-1^, 18 weeks		Sun and Palmer, 2018
Poaceae	*Eragrostis* sp*ectabilis*	5 dS·m^-1^, 65 days	Marked reduction in stomatal conductance may limit CO_2_ uptake, potentially affecting growth over extended saline periods	Wang et al., 2019b
Poaceae	*Leymus arenarius*	10 dS·m^-1^, 18 weeks		Sun and Palmer, 2018
Poaceae	*Miscanthus sinensis*	10 dS·m^-1^, 65 days		Wang et al., 2019b
Poaceae	*Schizachyrium scoparium*	10 dS·m^-1^, 65 days	High reductions in photosynthesis and stomatal conductance suggest limited carbon assimilation and gas exchange, potentially leading to future growth inhibition	Wang et al., 2019b
Poaceae	*Zoysia matrella*	10 dS·m^-1^, 8 weeks		Hooks et al., 2022
Poaceae	*Zoysia japonica*	10 dS·m^-1^, 8 weeks		Hooks et al., 2022
Portulacaceae	*Portulaca grandiflora*	3.2-4.5 dS·m^-1^, 8 weeks to 3 months		Devitt and Morris, 1987; Gupta et al., 2018
Ranunculaceae	*Anemone coronaria*	4.5 dS·m^-1^, 8 weeks	Browning on the edge and the middle of leaves	Rauter et al., 2021
Verbenaceae	*Glandularia canadensis*	3.2 dS·m^-1^, 103 days	Foliage injuries observed	Niu et al., 2007
Verbenaceae	*Lantana ×hybrida*	5.4 dS·m^-1^, 103 days		Niu et al., 2007
Verbenaceae	*Verbena ×hybrida*	5 dS·m^-1^, 8 weeks		Sun et al., 2015a
Verbenaceae	*Verbena macdougalii*	5.4 dS·m^-1^, 103 days		Niu et al., 2007

i threshold classification is defined as no more than 25% foliage injury or growth reduction observed, moderated from the method of Miyamoto et al. (2004).

^ii^the electricity conductivity (EC) and the duration of saline irrigation.

It is noteworthy that the 2019 Census of Horticultural Specialties includes numerous economically important ornamentals. However, some, such as Pentas spp. and Thunbergia spp., have not yet been studied for salinity tolerance in scientific literature. The absence of empirical data on these widely cultivated species highlights a research gap that warrants future investigation. Moreover, while flower quantity is often measured, comprehensive evaluation of flower quality traits, including color, fragrance, and texture, are rarely included in salt-tolerance evaluations. Future studies should prioritize evaluating untested but commercially relevant ornamentals and developing standardized criteria for flower quality under saline conditions. In addition, research should explore physiological mechanisms underlying salinity resilience to support breeding and selection of salt-tolerant species.

## Data Availability

The datasets presented in this article are not readily available because the ethical approval for this study does not allow raw sequencing data to be uploaded into a data repository. Requests to access the datasets should be directed to corresponding author.
