# Supplementary material for: Response of ornamental plants to salinity: impact on species-specific growth, visual quality, photosynthetic parameters, and ion uptake
Source: Front Plant Sci. 2025 Jul 30;16:1611767. doi: 10.3389/fpls.2025.1611767 (PMC12343571; doi:10.3389/fpls.2025.1611767)
Supplement: Supplementary file 1 [file Supplementaryfile1.docx]

Alpha, C. G., Drake, D. R., and Goldstein, (1996). Morphological and physiological responses of Scaevola sericea (Goodeniaceae) seedlings to salt spray and substrate salinity. *Am. J. Botany.* 83, 86-92. doi: 10.1002/j.1537-2197.1996.tb13878.x

Azizi, M., Chehrazi, M., and Zahedi, S. M. (2011). Effects of salinity stress on germination and early growth of sweet William (*Dianthus barbatus*). *Asian J. Agric. Sci.* 3, 453-458. Available online at: <https://www.researchgate.net/publication/267963608>. [accessed Nov 15, 2011]

Cristiano, G., Vuksani, G., Tufarelli, V., and De Lucia, B. (2018). Response of weeping lantana (*Lantana montevidensis*) to compost-based growing media and electrical conductivity level in soilless culture: first evidence. *Plants*. 7, 24. doi: 10.3390/plants7020024

Niu, G., Rodriguez, D. S., and Starman, T. (2010b). Response of bedding plants to saline water irrigation. *HortScience.* 45, 628-636. doi: 10.21273/HORTSCI.45.4.628

Pušić, M. G., Mladenovic, E. M., Cukanovic, J. D., Lakicevic, M. D., and Pavlovic,L. M. (2019). Influence of salinity on the growth and development of pansies (*Viola x wittrockiana* Gams.). *Zbornik Matice Srpske za Prirodne Nauke*. 137, 57-66. doi: 10.2298/ZMSPN1937057P

Tok, S., and Temizel, K. E. (2022). Effects of irrigation water in different salinity on yield and quality parameters of tobacco (*Nicotiana tabacum* L.) plant. *Gesunde Pflanzen*. 74, 9-16. doi: 10.1007/s10343-021-00584-0

Tomar, O. S., and Minhas, P. S. (2002). Performance of winter annual flowering species as affected by different modes of saline and canal water irrigation. *Indian J. Horticulture*. 59, 201-206. Available online at: https://www.Indianjournals.com/ijor. aspx?target=ijor:ijh&volume=59&issue=2&article=020&type=pdf. [accessed Jan, 2002]

Wahocho, N. A., Laghari, R. M. U. R., Talpur, K. H., Jamali, M. F., Ahmad, W., Shah, A. N., et al. (2023). Seed germination and vegetative growth of petunia (*Petunia hybrida*) genotypes to salt stress. *J. Appl. Res. Plant Sci.* 4, 553-565. doi: 10.38211/joarps.2023.04.02.173
