# Supplementary material for: Response of ornamental plants to salinity: impact on species-specific growth, visual quality, photosynthetic parameters, and ion uptake
Source: Front Plant Sci. 2025 Jul 30;16:1611767. doi: 10.3389/fpls.2025.1611767 (PMC12343571; doi:10.3389/fpls.2025.1611767)
Supplement: Supplementary file 2 [file Table1.docx]

Supplementary Table 1. Effects of saline irrigation on aesthetic performance in different ornamental species

| **Botanical family** | **Species** | **Salt treatments** | **Visual observations** | **References** |
| --- | --- | --- | --- | --- |
| Acanthaceae | *Anisacanthus quadrifidus* | 5-10 dS·m^-1 i^, 8 weeks^ii^ | Excellent performance at EC5; slight damage at EC10 | Wu et al. 2016a |
| Acanthaceae | *Dicliptera suberecta* | 10 dS·m^-1^, 8 weeks | Excellent performance | Wu et al. 2016a |
| Acanthaceae | *Ruellia brittoniana* | 10 dS·m^-1^, 8 weeks | Good quality with minimal damage | Sun et al. 2015a |
| Acoraceae | *Acorus gramineus* | 5-10 dS·m^-1^, 95 days | ~50% salt damage at EC5; all dead at EC10 | Xing et al. 2021 |
| Adoxaceae | *Viburnum×burkwoodii* | 5-10 dS·m^-1^, 8 weeks | Excellent quality at EC5; ~50% damage at EC10 | Sun et al. 2020 |
| Adoxaceae | *Viburnum cassinoides* | 5-10 dS·m^-1^, 8 weeks | ~50% damage at EC5; >90% damage at EC10 | Sun et al. 2020 |
| Adoxaceae | *Viburnum dentatum* | 5-10 dS·m^-1^, 8 weeks | Good quality with minimal damage at EC5; >90% damage at EC10 with high mortality | Sun et al. 2020 |
| Adoxaceae | *Viburnum dilatatum* | 5-10 dS·m^-1^, 8 weeks | >90% damage at EC5; all dead at EC10 | Sun et al. 2020 |
| Adoxaceae | *Viburnum×‘NCVX1’* | 5-10 dS·m^-1^, 8 weeks | Good quality with minimal damage at EC5; <50% damage at EC10 | Sun et al. 2020 |
| Adoxaceae | *Viburnum nudum* | 5-10 dS·m^-1^, 8 weeks | Good quality with minimal damage at EC5; >90% damage at EC10 | Sun et al. 2020 |
| Adoxaceae | *Viburnum opulus* | 5-10 dS·m^-1^, 8 weeks | ~50% damage at EC5; >90% damage at EC10 | Sun et al. 2020 |
| Adoxaceae | *Viburnum plicatum* | 5-10 dS·m^-1^, 8 weeks | >50% damage at EC5; all dead at EC10 | Sun et al. 2020 |
| Adoxaceae | *Viburnum pragense* | 5-10 dS·m^-1^, 8 weeks | Good quality with minimal damage at EC5; >90% damage at EC10 | Sun et al. 2020 |
| Adoxaceae | *Viburnum×rhytidophylloides* | 5-10 dS·m^-1^, 8 weeks | Good quality with minimal damage at EC5; >90% damage at EC10 | Sun et al. 2020 |
| Adoxaceae | *Viburnum trilobum* | 5-10 dS·m^-1^, 8 weeks | >90% damage at EC5; all dead at EC10 | Sun et al. 2020 |
| Asteraceae | *Achillea millefolium* | 5.4 dS·m^-1^, 103 days | Not affected | Niu et al. 2007 |
| Asteraceae | *Achillea millefolium* | 4 dS·m^-1^, 10 weeks | Good quality with minimal foliage salt damage | Niu and Rodriguez 2006a |
| Asteraceae | *Chrysactinia mexicana* | 10 dS·m^-1^, 5 weeks | ~50% salt damage | Wu et al. 2016b |
| Asteraceae | *Echinacea purpurea* | 2 dS·m^-1^, 10 weeks | Unacceptable performance | Niu and Rodriguez 2006a |
| Asteraceae | *Eupatorium greggii* | 10 dS·m^-1^, 5 weeks | Good quality with minimal foliage salt damage | Wu et al. 2016b |
| Asteraceae | *Gaillardia aristata* | 2 dS·m^-1^, 10 weeks | Good quality with minimal foliage salt damage | Niu and Rodriguez 2006a |
| Asteraceae | *Gaillardia aristata* | 5.4 dS·m^-1^, 103 days | Not affected | Niu et al. 2007 |
| Asteraceae | *Gazania rigen* | 12 dS·m^-1^, 12 weeks | Good quality with minimal foliage salt damage | Niu and Rodriguez 2006b |
| Asteraceae | *Gerbera jamesonii* | ~4.6 dS·m^-1^, 5 months | Older leaf senescence | Don et al. 2010 |
| Asteraceae | *Gerbera jamesonii* | ~12.9 dS·m^-1^, 20 days | Bleaching | Uzma et al. 2022a |
| Asteraceae | *Leucanthemum ×superbum* | 10 dS·m^-1^, 5 weeks | ~50% salt damage | Wu et al. 2016b |
| Asteraceae | *Melampodium leucanthum* | 5-10 dS·m^-1^, 5 weeks | Good quality at EC5; 50%-90% damage at EC10 | Wu et al. 2016b |
| Asteraceae | *Osteospermum hybrida* | 5 dS·m^-1^, 82 days | Basal leaves damaged | Valdés et al. 2015 |
| Asteraceae | *Rudbeckia fulgida* | 16.1 dS·m^-1^, 6 weeks | Chlorosis, foliar and crown death | Gerber et al. 2011 |
| Asteraceae | *Rudbeckia hirta* | 5.4 dS·m^-1^, 103 days | Severe salt damage | Niu et al. 2007 |
| Asteraceae | *Santolina chamaecyparissus* | 10 dS·m^-1^, 5 weeks | Good quality with minimal damage | Wu et al. 2016b |
| Asteraceae | *Symphyotrichum oblongifolium* | 10 dS·m^-1^, 5 weeks | >50% salt damage | Wu et al. 2016b |
| Asteraceae | *Tagetes erecta* | 3-6 dS·m^-1^, 8 weeks | Minimal to moderate damage at EC3; moderate to ~90% damage at EC6 | Sun et al. 2018b |
| Asteraceae | *Tagetes lemmonii* | 5-10 dS·m^-1^, 5 weeks | Good quality at EC5; 50%-90% damage at EC10 | Wu et al. 2016b |
| Asteraceae | *Tagetes patula* | 3-6 dS·m^-1^, 8 weeks | Good quality to slight damage at EC3; >90% damage at EC6 with high mortality | Sun et al. 2018b |
| Asteraceae | *Tetraneuris scaposa* | 5-10 dS·m^-1^, 5 weeks | 50%-90% damage at EC5; >90% damage at EC10 | Wu et al. 2016b |
| Asteraceae | *Viguiera stenoloba* | 5-10 dS·m^-1^, 5 weeks | Excellent performance at EC5; good quality with minimal damage at EC10 | Wu et al. 2016b |
| Asteraceae | *Wedelia texana* | 5-10 dS·m^-1^, 5 weeks | ~50% damage at EC5; 50%-90% damage at EC10 | Wu et al. 2016b |
| Asteraceae | *Zinnia angustifolia* | ~2.6 dS·m^-1^, 5 weeks | Marginal necrosis on lower leaves | Villarino and Mattson 2011 |
| Aizoaceae | *Delosperma cooperi* | 12 dS·m^-1^, 12 weeks | Good quality with minimal foliage salt damage | Niu and Rodriguez 2006b |
| Balsaminaceae | *Impatiens walleriana* | 3.9 dS·m^-1^ | Leave wilting and chlorosis | Roozbahani et al. 2020 |
| Brassicaceae | *Brassica oleracea* | ~6.4 dS·m^-1^, 15 days | No significant damage | Salachna et al. 2017 |
| Campanulaceae | *Lobelia cardinalis* | 5-10 dS·m^-1^, 8 weeks | ~50% damage at EC5; 50%-90% damage at EC10 | Wu et al. 2016a |
| Campanulaceae | *Lobelia erinus* | 2 dS·m^-1^, 60 days | No significant effect | Escalona et al. 2013 |
| Caprifoliacea | *Diervilla rivularis* | 5-10 dS·m^-1^, 8 weeks | ~90% damage at EC5; all dead at EC10 | Liu et al. 2017 |
| Caprifoliaceae | *Lonicera japonica* | 5.4 dS·m^-1^, 103 days | Not affected | Niu et al. 2007 |
| Caprifoliaceae | *Scabiosa columbaria* | 5-10 dS·m^-1^, 8 weeks | <50% damage at EC5; 50%-90% damage at EC10 | Wu et al. 2016a |
| Cleomaceae | *Cleome gynandra* | ~11 dS·m^-1^, 5 weeks | Severe salt damage | Mwai et al. 2002 |
| Convolvulaceae | *Evolvulus glomeratus* | 10 dS·m^-1^, 8 weeks | Good quality with minimal damage | Hooks and Niu 2019 |
| Cornaceae | *Cornus alba* | 5-10 dS·m^-1^, 8 weeks | Slight to severe damage at EC5; all dead at EC10 | Liu et al. 2020 |
| Crassulaceae | *Sedum telephium* | 10 dS·m^-1^, 8 weeks | Minimal to slight salt damage | Hooks and Niu 2019 |
| Crassulaceae | *Sedum reflexum* | 10 dS·m^-1^, 8 weeks | Good quality with minimal damage | Hooks and Niu 2019 |
| Crassulaceae | *Sedum rupestre* | 10 dS·m^-1^, 8 weeks | Minimal to slight salt damage | Hooks and Niu 2019 |
| Cyperaceae | *Carex morrowii* | 5-10 dS·m^-1^, 95 days | ~50% damage at EC5; <90% damage at EC10 | Xing et al. 2021 |
| Cyperaceae | *Carex vulpinoidea* | 10 dS·m^-1^, 8 weeks | Moderate salt damage | Sun and Palmer 2018 |
| Elaeagnaceae | *Shepherdia ×utahensis* | 10 dS·m^-1^, 8 weeks | Excellent quality | Paudel and Sun 2023 |
| Ericaceae | *Arctostaphylos uva-ursi* | 5-10 dS·m^-1^, 8 weeks | Minimal damage at EC5; all dead at EC10 | Paudel and Sun 2023 |
| Euphorbiaceae | *Jatropha curcas* | 9 dS·m^-1^, 54 days | 30% leaves edge yellowing | Niu et al. 2012b |
| Fabaceae | *Albizia julibrissin* | 10 dS·m^-1^, 8 weeks | Good quality with minimal damage | Paudel and Sun 2022 |
| Fabaceae | *Cercis canadensis* | 6 dS·m^-1^, 167 days | Marginal burn and leaf curling | Niu et al. 2010a |
| Fabaceae | *Sophora japonica* | 10 dS·m^-1^, 8 weeks | Good quality with minimal damage | Paudel and Sun 2022 |
| Fabaceae | *Sophora secundiflora* | 6 dS·m^-1^, 194 days | Not affected | Niu et al. 2010a |
| Geraniaceae | *Pelargonium ×hortorum* | 6.5 dS·m^-1^, 88 days | 21% leaf damage | Valdés et al. 2015 |
| Hydrangeaceae | *Dichroa febrifuga ×Hydrangea macrophylla* | 10 dS·m^-1^, 52 days | Good quality with minimal damage to moderate damage | Sun et al. 2022 |
| Hydrangeaceae | *Hydrangea macrophylla* | 5-10 dS·m^-1^, 4 weeks | Minimal damage at EC5; slight to severe damage at EC10 | Niu et al. 2020 |
| Hydrangeaceae | *Hydrangea macrophylla* | 5-10 dS·m^-1^, 8 weeks | Good quality with minimal damage at EC5; ~50% damage at EC10 | Liu et al. 2017 |
| Hydrangeaceae | *Hydrangea paniculata* | 5-10 dS·m^-1^, 4 weeks | Minimal to severe damage at EC5; >90% damage at EC10 | Niu et al. 2020 |
| Hydrangeaceae | *Hydrangea quercifolia* | 5-10 dS·m^-1^, 4 weeks | Good quality at EC5; >90% damage at EC10 | Niu et al. 2020 |
| Hydrangeaceae | *Hydrangea serrata* | 5-10 dS·m^-1^, 4 weeks | ~50% damage at EC5; >90% damage at EC10 | Niu et al. 2020 |
| Hydrangeaceae | *Hydrangea serrata ×macrophylla* | 5-10 dS·m^-1^, 4 weeks | Good quality with minimal damage | Niu et al. 2020 |
| Lamiaceae | *Agastache cana* | 2 dS·m^-1^, 10 weeks | Unacceptable performance | Niu and Rodriguez 2006a |
| Lamiaceae | *Ajuga reptans* | 5-10 dS·m^-1^, 6 weeks | Good quality with minimal damage at EC5; all dead at EC10 | Wu et al. 2016c |
| Lamiaceae | *Caryopteris ×clandonensis* | 5-10 dS·m^-1^, 8 weeks | Excellent performance at EC5; ~50% damage at EC10 | Wu et al. 2016a |
| Lamiaceae | *Lamium maculatum* | 5-10 dS·m^-1^, 6 weeks | ~50% leaf damage at EC5; 50%-90% damage at EC10 | Wu et al. 2016c |
| Lamiaceae | *Lavandula angustifolia* | 3.2-6.4 dS·m^-1^, 10 weeks | > 50% leaf damage at EC3.2; all plants dead at EC6.4 | Niu and Rodriguez 2006b |
| Lamiaceae | *Perovskia atriplicifolia* | 5-10 dS·m^-1^, 6 weeks | Good quality with minimal damage at EC5; ~50% damage at EC10 | Wu et al. 2016c |
| Lamiaceae | *Poliomintha longiflora* | 5-10 dS·m^-1^, 6 weeks | 50%-90% moderate damage at EC5; all dead at EC10 | Wu et al. 2016c |
| Lamiaceae | *Rosmarinus officinalis* | 5.4 dS·m^-1^, 103 days | Not affected | Niu et al. 2007 |
| Lamiaceae | *Salvia coccinea* | 4 dS·m^-1^, 10 weeks | Good quality with minimal foliage salt damage | Niu and Rodriguez 2006a |
| Lamiaceae | *Salvia farinacea* | 10 dS·m^-1^, 8 weeks | Good quality with minimal damage | Sun et al. 2015a |
| Lamiaceae | *Salvia leucantha* | 5-10 dS·m^-1^, 8 weeks | Excellent performance at EC5; 50%-90% damage at EC10 | Sun et al. 2015a |
| Lamiaceae | *Scutellaria suffrutescens* | 5-10 dS·m^-1^, 6 weeks | 50%-90% moderate damage at EC5; all dead at EC10 | Wu et al. 2016c |
| Lamiaceae | *Stachys coccinea* | 10 dS·m^-1^, 6 weeks | Good quality with minimal damage | Wu et al. 2016c |
| Lamiaceae | *Teucrium chamaedrys* | 12 dS·m^-1^, 12 weeks | Slight salt damage (< 50%) | Niu and Rodriguez 2006b |
| Lythraceae | *Cuphea hyssopifolia* | 5-10 dS·m^-1^, 8 weeks | Good quality minimal damage at EC5; 50%-90% damage at EC10 | Wu et al. 2016a |
| Malvaceae | *Hibiscus syriacus* | 6.5 dS·m^-1^, 11 weeks | No salt damage | Chen et al. 2019a |
| Malvaceae | *Hibiscus syriacus* | 5-10 dS·m^-1^, 8 weeks | >50% damage at EC5; >90% damage at EC10 | Liu et al. 2017 |
| Malvaceae | *Malvaviscus arboreus* | 10 dS·m^-1^, 8 weeks | Excellent performance | Sun et al. 2015a |
| Malvaceae | *Pavonia lasiopetala* | 5-10 dS·m^-1^, 8 weeks | Good quality minimal damage at EC5; <50% damage at EC10 | Wu et al. 2016a |
| Oleaceae | *Forsythia ×intermedia* | 5-10 dS·m^-1^, 8 weeks | Good quality with minimal damage at EC5; ~50% damage at EC10 | Liu et al. 2017 |
| Plantaginaceae | *Angelonia angustifolia* | 7.4 dS·m^-1^, 122 days | Excellent performance | Niu et al. 2010b |
| Plantaginaceae | *Penstemon barbatus* | 5-10 dS·m^-1^, 8 weeks | ~50% salt damage at EC5; ~90% damage at EC7.5 and EC10 | Paudel and Sun 2024 |
| Plantaginaceae | *Penstemon davidsonii* | 2.5-10 dS·m^-1^, 8 weeks | Slight damage at EC5; >90% leaf damage at EC7.5; all plants dead at EC10 | Nepal et al. 2024 |
| Plantaginaceae | *Penstemon eatonii* | 12 dS·m^-1^, 10 weeks | 50-90% leaf salt damage | Niu and Rodriguez 2006b |
| Plantaginaceae | *Penstemon heterophyllus* | 2.5-10 dS·m^-1^, 8 weeks | Good quality with minimal damage at EC5; 50%-90% damage at EC7.5; >90% damage at EC10 | Nepal et al. 2024 |
| Plantaginaceae | *Penstemon pseudospectabili* | 3.2-6.4 dS·m^-1^, 10 weeks | 50-90% leaf salt damage at EC3.2; all plants dead at EC6.4 | Niu and Rodriguez 2006b |
| Plantaginaceae | *Penstemon strictus* | 3.2-6.4 dS·m^-1^, 10 weeks | 50-90% leaf salt damage at EC3.2; all plants dead at EC6.4 | Niu and Rodriguez 2006b |
| Plantaginaceae | *Penstemon strictus* | 2.5-10 dS·m^-1^, 8 weeks | Good quality with minimal damage at EC5; >50% damage at EC 7.5; ~90% damage at EC10 | Paudel and Sun 2024 |
| Plumbaginaceae | *Ceratostigma plumbaginoides* | 3.2-12 dS·m^-1^, 12 weeks | ~50% leaves damage at EC3.2; ~90% leaves damage at EC12 | Niu and Rodriguez 2006b |
| Poaceae | *Andropogon ternarius* | 10 dS·m^-1^, 95 days | >50% salt damage | Xing et al. 2021 |
| Poaceae | *Calamagrostis ×acutiflora* | 10 dS·m^-1^, 95 days | Excellent performance | Xing et al. 2021 |
| Poaceae | *Chasmanthium latifolium* | 10 dS·m^-1^, 18 weeks | Minimal damage | Sun and Palmer 2018 |
| Poaceae | *Eragrostis spectabilis* | 10 dS·m^-1^, 65 days | Slight salt damage | Wang et al. 2019b |
| Poaceae | *Festuca glauca* | 5-10 dS·m^-1^, 95 days | Good quality at EC5; ~50% leaf damage at EC10 | Xing et al. 2021 |
| Poaceae | *Leymus arenarius* | 10 dS·m^-1^, 18 weeks | Minimal damage | Sun and Palmer 2018 |
| Poaceae | *Miscanthus sinensis* | 10 dS·m^-1^, 65 days | Slight salt damage | Wang et al. 2019b |
| Poaceae | *Muhlenbergia capillaris* | 5-10 dS·m^-1^, 18 weeks | No foliage damage at EC5; minimal to slight damage at EC10 | Sun and Palmer 2018 |
| Poaceae | *Panicum virgatum* | 10 dS·m^-1^, 65 days | Not affected | Wang et al. 2019b |
| Poaceae | *Pennisetum alopecuroides* | 5-10 dS·m^-1^, 18 weeks | No foliage damage at EC5; minimal to slight damage at EC10 | Sun and Palmer 2018 |
| Poaceae | *Schizachyrium scoparium* | 10 dS·m^-1^, 65 days | Not affected | Wang et al. 2019b |
| Poaceae | *Sporobolus heterolepis* | 5-10 dS·m^-1^, 95 days | Excellent quality at EC5; ~50% leaf damage at EC10 | Xing et al. 2021 |
| Poaceae | *Zoysia matrella* | 10 dS·m^-1^, 8 weeks | Not affected | Hooks et al. 2022 |
| Poaceae | *Zoysia minima* | 10 dS·m^-1^, 8 weeks | Not affected | Hooks et al. 2022 |
| Poaceae | *Zoysia japonica* | 10 dS·m^-1^, 8 weeks | Not affected | Hooks et al. 2022 |
| Polemoniaceae | *Phlox paniculata* | 5-10 dS·m^-1^, 8 weeks | >50% damage at EC5; all dead at EC10 | Sun et al. 2015a |
| Ranunculaceae | *Anemone coronaria* | 4.5 dS·m^-1^, 8 weeks | Browning on the edge and the middle of leaves | Rauter et al. 2021 |
| Ranunculaceae | *Aquilegia canadensis* | 5-10 dS·m^-1^, 8 weeks | >50% damage at EC5; almost all dead at EC10 | Wu et al. 2016c |
| Ranunculaceae | *Ranunculus asiaticus* | 5.5 dS·m^-1^, 8 weeks | Browning on the edge and the middle of leaves | Rauter et al. 2021 |
| Rosaceae | *Cercocarpus ledifolius* | 10 dS·m^-1^, 8 weeks | ~50% leaves damage | Paudel and Sun 2023 |
| Rosaceae | *Cercocarpus montanus* | 5-10 dS·m^-1^, 8 weeks | ~50% leaves damage at EC5; all dead at EC10 | Paudel and Sun 2023 |
| Rosaceae | *Chaenomeles speciosa* | 5-10 dS·m^-1^, 8 weeks | 50%-90% damage at EC5; all dead at EC10 | Liu et al. 2017 |
| Rosaceae | *Physocarpus opulifolius* | 5.2 dS·m^-1^, 11 weeks | ~50% salt damage | Chen et al. 2019a |
| Rosaceae | *Spiraea japonica* | 3 dS·m^-1^, 8 weeks | Good quality with slight damage | Wang et al. 2019a |
| Rosaceae | *Spiraea japonica* | 3.4 dS·m^-1^, 11 weeks | ~50% salt damage | Chen et al. 2019a |
| Rosaceae | *Rosa fortuniana* | 9 dS·m^-1^, 15 weeks | Foliage injuries observed | Niu et al. 2008 |
| Rosaceae | *Rosa multiflora* | 9 dS·m^-1^, 15 weeks | Severe damage, especially on lower leaves | Niu et al. 2008 |
| Rosaceae | *Rosa odorata* | 9 dS·m^-1^, 15 weeks | Severe damage, especially on lower leaves | Niu et al. 2008 |
| Rosaceae | *Rosa spp.* | 6.4 dS·m^-1^, 7 weeks | Excellent without foliage salt damage | Niu et al. 2013 |
| Rosaceae | *Rosa spp.* | 6.4 dS·m^-1^, 10 weeks | ‘Belinda’s Dream’, ‘Caldwell Pink’, and ‘Quietness’: excellent or good quality with minimal foliage salt damage; ‘Carefree Beauty’, ‘Folksinger’, and ‘Winter Sunset’: >90% leaves burn. | Niu et al. 2013 |
| Rosaceae | *Rosa spp.* | 10 dS·m^-1^, 43 days | Minimal to ~90% salt damage | Cai et al. 2014a |
| Solanaceae | *Capsicum annuum* | 8.1 dS·m^-1^, 57 days | Minor foliage damage | Niu et al. 2012a |
| Solanaceae | *Capsicum annuum* | 4.1 dS·m^-1^, 74 days | Good performance | Niu et al. 2010c |
| Solanaceae | *Cestrum spp.* | 10 dS·m^-1^, 8 weeks | Excellent performance | Wu et al. 2016a |
| Verbenaceae | *Glandularia canadensis* | 5.4 dS·m^-1^, 103 days | Foliage injuries observed | Niu et al. 2007 |
| Verbenaceae | *Glandularia ×hybrida* | 5.4 dS·m^-1^, 103 days | Not affected | Niu et al. 2007 |
| Verbenaceae | *Lantana camara* | 5.1 dS·m^-1^, 175 days | 25%-35% leaf discoloration, leaf necrosis and 15%-20% defoliation | Bañón et al. 2011 |
| Verbenaceae | *Lantana montevidensis* | 5.4 dS·m^-1^, 103 days | Severe salt damage | Niu et al. 2007 |
| Verbenaceae | *Lantana ×hybrida* | 5.4 dS·m^-1^, 103 days | Not affected | Niu et al. 2007 |
| Verbenaceae | *Verbena ×hybrida* | 10 dS·m^-1^, 8 weeks | Good quality with minimal damage | Sun et al. 2015a |
| Verbenaceae | *Verbena macdougalii* | 5.4 dS·m^-1^, 103 days | Not affected | Niu et al. 2007 |
| Vitaceae | *Parthenocissus quinquefolia* | 5-10 dS·m^-1^, 8 weeks | Excellent performance at EC5; ~50% damage at EC10 | Liu et al. 2017 |

^i^ the electricity conductivity (EC) of saline irrigation

^ii^ the duration of saline irrigation
