# Supplementary material for: Response of ornamental plants to salinity: impact on species-specific growth, visual quality, photosynthetic parameters, and ion uptake
Source: Front Plant Sci. 2025 Jul 30;16:1611767. doi: 10.3389/fpls.2025.1611767 (PMC12343571; doi:10.3389/fpls.2025.1611767)
Supplement: Supplementary file 3 [file Table2.docx]

Supplementary Table 2. Effects of saline irrigation on growth parameters, including dry weight (DW), plant height, leaf area, flower number, in different ornamental species

| **Botanical family** | **Species** | **Salt treatments** | **Growth observations** | **References** |
| --- | --- | --- | --- | --- |
| Acanthaceae | *Anisacanthus quadrifidus* | 5-10 dS·m^-1 i^, 8 weeks^ii^ | Shoot DW, height, and leaf area reductions of 32%-62%, 11%-27%, and 50%-77%, respectively | Wu et al. 2016a |
| Acanthaceae | *Dicliptera suberecta* | 5-10 dS·m^-1^, 8 weeks | Shoot DW, height, and leaf area reductions of 24%-43%, 7%-17%, and 30%-52%, respectively | Wu et al. 2016a |
| Acanthaceae | *Ruellia brittoniana* | 5-10 dS·m^-1^, 8 weeks | Shoot DW and height of 18%-32% and 40%-49%, respectively | Sun et al. 2015a |
| Acoraceae | *Acorus gramineus* | 5-10 dS·m^-1^, 95 days | Height and leaf area reductions of 16%-20% and 27%-45%, respectively | Xing et al. 2021 |
| Adoxaceae | *Viburnum×burkwoodii* | 5-10 dS·m^-1^, 8 weeks | Shoot DW, height, leaf area reductions of 24%, 52%, and 31%-45%, respectively | Chen et al, 2020; Sun et al. 2020 |
| Adoxaceae | *Viburnum cassinoides* | 10 dS·m^-1^, 8 weeks | Height reduction of 71% | Sun et al. 2020 |
| Adoxaceae | *Viburnum dentatum* | 10 dS·m^-1^, 8 weeks | Shoot DW, height, leaf area reductions of 59%, 72%, and 58%, respectively | Sun et al. 2020 |
| Adoxaceae | *Viburnum dilatatum* | 5 dS·m^-1^, 8 weeks | Shoot DW reduction of 51% | Sun et al. 2020 |
| Adoxaceae | *Viburnum*×‘NCVX1’ | 10 dS·m^-1^, 8 weeks | Shoot DW reduction of 25% | Chen et al. 2020; Sun et al. 2020 |
| Adoxaceae | *Viburnum nudum* | 5-10 dS·m^-1^, 8 weeks | Shoot DW and height reductions of 69% and 30%-70%, respectively | Chen et al. 2020; Sun et al. 2020 |
| Adoxaceae | *Viburnum opulus* | 10 dS·m^-1^, 8 weeks | Shoot DW and height reductions of 37% and 79%, respectively | Sun et al. 2020 |
| Adoxaceae | *Viburnum plicatum* | 5 dS·m^-1^, 8 weeks | Leaf area reduction of 32% | Sun et al. 2020 |
| Adoxaceae | *Viburnum pragense* | 10 dS·m^-1^, 8 weeks | Shoot DW, height, and leaf area reductions of 56%, 60% and 63%, respectively | Chen et al. 2020; Sun et al. 2020 |
| Adoxaceae | *Viburnum×rhytidophylloides* | 5-10 dS·m^-1^, 8 weeks | Shoot DW, height, and leaf area reductions of 38%-73%, 50%-76% and 44%-84%, respectively | Chen et al. 2020; Sun et al. 2020 |
| Adoxaceae | *Viburnum trilobum* | 5 dS·m^-1^, 8 weeks | Height and leaf area reductions of 65% and 73%, respectively | Sun et al. 2020 |
| Amaranthaceae | *Celosia argentea* | 4.5 dS·m^-1^, 8 weeks | Shoot DW, height, and flower reductions of 23%, 34%, and 57%, respectively | Devitt and Morris 1987 |
| Amaranthaceae | *Celosia argentea* | 6 dS·m^-1^, 47 days | Shoot DW, height, and flower reductions of 61%, 39%, and 75%, respectively | Bezerra et al. 2020 |
| Amaranthaceae | *Celosia argentea* | ~7.7 dS·m^-1^ | Shoot DW, height, flower, and #shoots reductions of 59%, 26%, 45%, and 9%, respectively | Gholamzadeh Alam et al. 2022 |
| Amaranthaceae | *Gomphrena globosa* | 3.2 dS·m^-1^, 3 months | Height, #shoots, and #leaves reductions of 61%, 53%, and 66%, respectively | Gupta et al. 2018 |
| Apocynaceae | *Catharanthus roseus* | 4.7 dS·m^-1^, 4 months | Leaf DW, height, and leaf area reductions of 70%, 18%, and 44%, respectively | Mohammadi Kabari et al. 2024 |
| Apocynaceae | *Catharanthus roseus* | 6 dS·m^-1^, 47 days | Shoot DW, height, and #flowers reductions of 79%, 51%, and 82%, respectively | Bezerra et al. 2020 |
| Apocynaceae | *Catharanthus roseus* | 8.1 dS·m^-1^, 50 days | Linear decrease on shoot DW | Cartmill et al. 2013 |
| Asteraceae | *Achillea millefolium* | 5.4 dS·m^-1^, 103 days | Shoot FW not affected | Niu et al. 2007 |
| Asteraceae | *Achillea millefolium* | 2 dS·m^-1^, 10 weeks | Significant reduction on shoot DW; height not affected | Niu and Rodriguez 2006a |
| Asteraceae | *Ageratum conyzoides* | ~7.9 dS·m^-1^, 4 weeks | Significant reduction on height | Putri et al. 2024 |
| Asteraceae | *Ageratum conyzoides* | ~1.6-9.7 dS·m^-1^, 20 days | Shoot DW and leaf area not affected | Sun et al. 2012 |
| Asteraceae | *Ageratum houstonianum* | 4.5 dS·m^-1^, 8 weeks | Shoot DW, height, and flower reductions of 42%, 18%, and 82%, respectively | Devitt and Morris 1987 |
| Asteraceae | *Calendula officinalis* | ~4.7 dS·m^-1^, 137 days | Shoot DW, height, leaf area, flower, and #shoots reductions of 26%, 28%, 49%, 24% and 43%, respectively | Swaefy and Elziat 2020 |
| Asteraceae | *Calendula officinalis* | 9.7 dS·m^-1^, 4 weeks | Stem length reduction of 40% | Kozminska et al. 2017 |
| Asteraceae | *Calendula officinalis* | 12.5 dS·m^-1^, 70 days | Shoot DW, flower, and #leaf reductions of 57%, 69%, and 36%, respectively | Fornes et al. 2007 |
| Asteraceae | *Chrysactinia mexicana* | 10 dS·m^-1^, 5 weeks | Shoot DW, height, flower reductions of 56%-64%, 25%-31%, 90%-91%, respectively | Wu et al. 2016b |
| Asteraceae | *Cosmos bipinnatus* | 4.5 dS·m^-1^, 8 weeks | Shoot DW, height, and flower reductions of 58%, 64%, and 100%, respectively | Devitt and Morris 1987 |
| Asteraceae | *Dahlia spp.* | 4-5 dS·m^-1^, 3 months | Height and flower weight reduction of 57% and 60%, respectively | Tomar and Minhas 2002 |
| Asteraceae | *Echinacea purpurea* | 4 dS·m^-1^, 10 weeks | Shoot DW and height not affected | Niu and Rodriguez 2006a |
| Asteraceae | *Eupatorium greggii* | 10 dS·m^-1^, 5 weeks | Shoot DW, height, flower reductions of 27%, 12%, and 28%, respectively | Wu et al. 2016b |
| Asteraceae | *Gaillardia aristata* | 2-4 dS·m^-1^, 10 weeks | Significant reduction on shoot DW and height at EC4 and EC2, respectively | Niu and Rodriguez 2006a |
| Asteraceae | *Gaillardia aristata* | 5.4 dS·m^-1^, 103 days | Shoot FW reduction of 40% | Niu et al. 2007 |
| Asteraceae | *Gazania rigen* | 3.2-12 dS·m^-1^, 12 weeks | Shoot DW reductions of 25%-50% | Niu and Rodriguez 2006b |
| Asteraceae | *Gazania splendens* | 7.5 dS·m^-1^, 60 days | DW reduction of 31% | García-Caparrós et al. 2016 |
| Asteraceae | *Gerbera jamesonii* | ~4.6 dS·m^-1^, 5 months | Shoot DW and flower reductions of 35%-40% and 100% | Don et al. 2010 |
| Asteraceae | *Leucanthemum ×superbum* | 5-10 dS·m^-1^, 5 weeks | Shoot DW and height reductions of 40%-59% and 44%-64%, respectively | Wu et al. 2016b |
| Asteraceae | *Marigold spp.* | 4.5 dS·m^-1^, 8 weeks | Shoot DW, height, and flower reductions of 27%, 15%, and 72%, respectively | Devitt and Morris 1987 |
| Asteraceae | *Marigold spp.* | 4-5 dS·m^-1^, 3 months | Height and flower weight reductions of 50% and 50%, respectively | Tomar and Minhas 2002 |
| Asteraceae | *Marigold spp.* | ~5.2 dS·m^-1^, 5 weeks | Significant reduction on DW and height | Villarino and Mattson 2011 |
| Asteraceae | *Melampodium leucanthum* | 5-10 dS·m^-1^, 5 weeks | Shoot DW reductions of 34%-54%, | Wu et al. 2016b |
| Asteraceae | *Osteospermum hybrida* | 5 dS·m^-1^, 82 days | Linearly reductions on shoot DW (0.95 g decrease/ EC unit), height (1.79 cm decrease/ EC unit) and leaf area (0.51 dm^2^ decrease/ EC unit) | Valdés et al. 2015 |
| Asteraceae | *Rudbeckia hirta* | 5.4 dS·m^-1^, 103 days | Shoot FW not affected | Niu et al. 2007 |
| Asteraceae | *Santolina chamaecyparissus* | 10 dS·m^-1^, 5 weeks | Shoot DW and height reductions of 17%-21% and 17%-22%, respectively | Wu et al. 2016b |
| Asteraceae | *Senecio cineraria* | 13 dS·m^-1^, 30 days | Shoot DW reduction of 20% | Saito et al. 2015 |
| Asteraceae | *Symphyotrichum oblongifolium* | 10 dS·m^-1^, 5 weeks | Shoot DW, height, and flower reductions of 41%, 27%, and 32%, respectively | Wu et al. 2016b |
| Asteraceae | *Tagetes erecta* | 3-6 dS·m^-1^, 8 weeks | Shoot DW, leaf area, and flower reductions of 27%, 56%-70%, and 28%-52%, respectively | Sun et al. 2018b |
| Asteraceae | *Tagetes lemmonii* | 5-10 dS·m^-1^, 5 weeks | Shoot DW, height, flower reductions of 42%-66%, 22%, and 51%-80%, respectively | Wu et al. 2016b |
| Asteraceae | *Tagetes patula* | 3-6 dS·m^-1^, 8 weeks | Shoot DW and flower reductions of 41% and 30%, respectively | Sun et al. 2018b |
| Asteraceae | *Tagetes patula* | 6 dS·m^-1^, 47 days | Shoot DW, height, and #flowers reductions of 47%, 56%, and 45%, respectively | Bezerra et al. 2020 |
| Asteraceae | *Tetraneuris scaposa* | 5-10 dS·m^-1^, 5 weeks | Shoot DW, height, and flower reductions of 67%, 17%-37%, and 50%-83%, respectively | Wu et al. 2016b |
| Asteraceae | *Viguiera stenoloba* | 5-10 dS·m^-1^, 5 weeks | Shoot DW and flower reduction of 26%-34% and 37%, respectively | Wu et al. 2016b |
| Asteraceae | *Wedelia texana* | 5-10 dS·m^-1^, 5 weeks | Shoot DW and height reductions of 31% and 13%-28%, respectively | Wu et al. 2016b |
| Asteraceae | *Zinnia angustifolia* | ~2.6 dS·m^-1^, 5 weeks | Significant reductions on DW and height | Villarino and Mattson 2011 |
| Asteraceae | *Zinnia elegans* | 4.5 dS·m^-1^, 8 weeks | Shoot DW, height, and #flowers reductions of 79%, 51%, and 100%, respectively | Devitt and Morris 1987 |
| Asteraceae | *Zinnia maritima* | 3-4.2 dS·m^-1^, 26 days | Shoot DW reductions of 30%-48%; height reductions of 12%-29% | Niu et al. 2012c |
| Asteraceae | *Zinnia marylandica* | 3-4.2 dS·m^-1^, 26 days | Shoot DW reductions of 11%-57%; height reductions up to 38% | Niu et al. 2012c |
| Aizoaceae | *Delosperma cooperi* | 12 dS·m^-1^, 12 weeks | Shoot DW reduction of 30% | Niu and Rodriguez 2006b |
| Balsaminaceae | *Impatiens walleriana* | 3.1 dS·m^-1^ | DW and height reductions of 74% and 36%, respectively | Kuehny and Morales 1998 |
| Balsaminaceae | *Impatiens walleriana* | 3.9 dS·m^-1^ | DW and height reductions of 45% and 46%, respectively | Roozbahani et al. 2020 |
| Begoniaceae | *Begonia hiemalis* | ~5.2 dS·m^-1^, 5 weeks | Significant reduction on shoot DW; height not affected | Villarino and Mattson 2011 |
| Begoniaceae | *Begonia semperflorens* | ~3.6 dS·m^-1^, 12 weeks | Shoot DW, shoot length, flower, and #shoots reductions of 31%, 44%, 51%, and 52%, respectively | Çiçek 2023 |
| Brassicaceae | *Alyssum murale* | ~3.2-6.4 dS·m^-1^, 21 days | Shoot DW not affected at EC3.2; but significantly reduced at EC6.4; seed germination not affected | Comino et al. 2005 |
| Brassicaceae | *Brassica oleracea* | ~3.2-51.5 dS·m^-1^, 15 days | Height reduction of 34% | Salachna et al. 2017 |
| Brassicaceae | *Brassica spp.* | ~12.9 dS·m^-1^, 24 hours | Root growth reduction of 80% | Pavlović et al. 2019 |
| Brassicaceae | *Nasturtium officinale* | 9.7 dS·m^-1^, 21 days | Leaf area, #leaves, leaf DW reductions of 73%, 55%, and 63%, respectively | Kaddour et al. 2013 |
| Caryophyllaceae | *Dianthus barbatus* | 8 dS·m^-1^, 7 days | Seedling DW and radicle length reductions of 82% and 65%, respectively | Azizi et al. 2011 |
| Caryophyllaceae | *Dianthus chinensis* | 4.5 dS·m^-1^, 8 weeks | Height and flower reductions of 10% and 15%, respectively; shoot DW not affetced | Devitt and Morris 1987 |
| Caryophyllaceae | *Dianthus chinensis* | 7.8 dS·m^-1^, 39 days | Shoot DW reduction of 68%; linearly decrease on height | Zhang et al. 2019 |
| Cannaceae | *Canna indica* | 5-20 dS·m^-1^, 20 days | Significant increase on DW at EC5; significant reduction on DW at EC10, EC15, and EC20. | Chen et al. 2019b |
| Campanulaceae | *Lobelia cardinalis* | 5-10 dS·m^-1^, 8 weeks | Shoot DW, height, and leaf area reductions of 53%-70%, 30%-62%, and 64%-95%, respectively | Wu et al. 2016a |
| Campanulaceae | *Lobelia erinus* | 2 dS·m^-1^, 60 days | Linearly decrease on DW, height (0.04 cm decrease/EC unit) and diameter (0.33 cm decrease/EC unit) | Escalona et al. 2013 |
| Caprifoliacea | *Diervilla rivularis* | 5 dS·m^-1^, 8 weeks | Shoot DW, height, and leaf area reductions of 67%-80%, 37%-83%, and 95%-99%, respectively | Liu et al. 2017 |
| Caprifoliaceae | *Lonicera japonica* | 5.4 dS·m^-1^, 103 days | Shoot FW not affected | Niu et al. 2007 |
| Caprifoliaceae | *Scabiosa columbaria* | 5-10 dS·m^-1^, 8 weeks | Shoot DW, height, leaf area and flower reductions of 16%-48%, 34%-51%, 37%-71%, and 37%-56%, respectively | Wu et al. 2016a |
| Cleomaceae | *Cleome gynandra* | ~6.9 dS·m^-1^, 5 weeks | Significant reduction on shoot DW | Mwai et al. 2002 |
| Convolvulaceae | *Evolvulus glomeratus* | 5-10 dS·m^-1^, 8 weeks | Shoot DW reductions of 28%-58% | Hooks and Niu 2019 |
| Convolvulaceae | *Ipomoea purpurea* | ~12.9 dS·m^-1^, 3 weeks | Plant growth and radicle length reductions of 30% and 38%, respectively; leaf DW not affected | Mircea et al. 2023 |
| Convolvulaceae | *Ipomoea tricolor* | ~12.9 dS·m^-1^, 3 weeks | Radicle length reduction of 13%; height and leaf DW not affected | Mircea et al. 2023 |
| Cornaceae | *Cornus alba* | 5-10 dS·m^-1^, 8 weeks | Shoot DW reduction of 55% at EC5; height and leaf area reductions of 51% and 89%, respectively, at EC10 | Liu et al. 2020 |
| Crassulaceae | *Sedum telephium* | 5-10 dS·m^-1^, 8 weeks | Shoot DW reductions of 47%-84% | Hooks and Niu 2019 |
| Crassulaceae | *Sedum reflexum* | 5-10 dS·m^-1^, 8 weeks | Shoot DW reductions of 73%-80% | Hooks and Niu 2019 |
| Crassulaceae | *Sedum rupestre* | 5-10 dS·m^-1^, 8 weeks | Shoot DW reductions of 56%-80% | Hooks and Niu 2019 |
| Cyperaceae | *Carex morrowii* | 5-10 dS·m^-1^, 95 days | Shoot DW, height, leaf area reductions of 36%-79%, 16%-45%, and 32%-76%, respectively | Xing et al. 2021 |
| Cyperaceae | *Carex vulpinoidea* | 10 dS·m^-1^, 8 weeks | Height and leaf area reductions of 13-36% and 29%, respectively | Sun and Palmer 2018 |
| Elaeagnaceae | *Shepherdia ×utahensis* | 10 dS·m^-1^, 8 weeks | Shoot DW reduction of 32%; leaf area not affected | Paudel and Sun 2023 |
| Ericaceae | *Arctostaphylos uva-ursi* | 5 dS·m^-1^, 8 weeks | Leaf area reduction of 52% | Paudel and Sun 2023 |
| Euphorbiaceae | *Euphorbia lathyris* | 10.3-43.5 dS·m^-1^, 20 days | Biomass increase when EC<18.7 dS·m^-1^; biomass reduction up to 40% when EC>18.7 dS·m^-1^ | Yang et al. 2013 |
| Euphorbiaceae | *Euphorbia milii* | 5 dS·m^-1^, 50 days | Shoot DW and stem diameter reductions of 53% and 20%, respectively | Santos et al. 2022 |
| Euphorbiaceae | *Jatropha curcas* | 3-9 dS·m^-1^, 54 days | Shoot DW of 28%-42%; significant reduction on height and leaf area | Niu et al. 2012b |
| Fabaceae | *Albizia julibrissin* | 5-10 dS·m^-1^, 8 weeks | Shoot DW, height, and leaf area reductions of 53%-58%, 38%-72%, and 35%, respectively | Paudel and Sun 2022 |
| Fabaceae | *Cercis canadensis* | 3-6 dS·m^-1^, 167 days | Shoot DW reductions of >50% | Niu et al. 2010a |
| Fabaceae | *Sophora japonica* | 5-10 dS·m^-1^, 8 weeks | Shoot DW, height, and leaf area reductions of 68%-71%, 30%-45%, and 44%, respectively | Paudel and Sun 2022 |
| Fabaceae | *Sophora secundiflora* | 3-6 dS·m^-1^, 194 days | Shoot DW reductions of 25%-46% | Niu et al. 2010a |
| Gentianaceae | *Lisianthus spp.* | 12 dS·m^-1^, until flowering | Markable flower quality and growing well when EC<8 dS·m^-1^ | Valdez-Aguilar et al. 2013 |
| Gentianaceae | *Lisianthus spp.* | 12 dS·m^-1^, until flowering | Markable flower quality and growing well when EC<7 dS·m^-1^ | Valdez-Aguilar et al. 2014 |
| Gentianaceae | *Lisianthus* *spp.* | 8.5 dS·m^-1^, 70 days | Linearly reduction on height | Ashrafi and Nejad 2018 |
| Geraniaceae | *Pelargonium ×hortorum* | 6.5 dS·m^-1^, 88 days | Shoot DW, height, leaf area, and flower reductions of 65%, 46%, 58%, and 59%, respectively | Valdés et al. 2015 |
| Geraniaceae | *Pelargonium graveolens* | 8.5 dS·m^-1^, 30 days | Biomass and height not affected | Chrysargyris et al. 2021 |
| Goodeniaceae | *Scaevola sericea* | 15.6 dS·m^-1^, 8 weeks | Shoot mass, leaf area, and #leaves reductions of 76%, 87%, and 67%, respectively | Alpha et al. 1996 |
| Goodeniaceae | *Scaevola sericea* | 15.6 dS·m^-1^, 8 weeks | Stem and leaf growth reduction of 65% | Goldstein et al. 1996 |
| Hydrangeaceae | *Dichroa febrifuga ×Hydrangea macrophylla* | 5-10 dS·m^-1^, 52 days | Shoot DW, height, and leaf area reductions of 35%-67%, 39%-94%, and 39%-91%, respectively | Sun et al. 2022 |
| Hydrangeaceae | *Hydrangea macrophylla* | 5-10 dS·m^-1^, 4 weeks | Shoot DW not affected; leaf area reductions of 20%-58% and 42%-89%, respectively, at EC5 and EC10, respectively | Niu et al. 2020 |
| Hydrangeaceae | *Hydrangea macrophylla* | 5-10 dS·m^-1^, 8 weeks | Shoot DW, height, and leaf area reductions of 54%-75%, 57%-93%, and 57%-88%, respectively | Liu et al. 2017 |
| Hydrangeaceae | *Hydrangea paniculata* | 5-10 dS·m^-1^, 4 weeks | Shoot DW not affected; leaf area reductions of 20%-58% and 42%-89%, respectively, at EC5 and EC10, respectively | Niu et al. 2020 |
| Hydrangeaceae | *Hydrangea quercifolia* | 5-10 dS·m^-1^, 4 weeks | Shoot DW not affected; leaf area reductions of 20%-58% and 42%-89%, respectively, at EC5 and EC10, respectively | Niu et al. 2020 |
| Hydrangeaceae | *Hydrangea serrata* | 5-10 dS·m^-1^, 4 weeks | Shoot DW not affected; leaf area reductions of 20%-58% and 42%-89%, respectively, at EC5 and EC10, respectively | Niu et al. 2020 |
| Hydrangeaceae | *Hydrangea serrata ×macrophylla* | 5-10 dS·m^-1^, 4 weeks | Shoot DW not affected; leaf area reductions of 20%-58% and 42%-89%, respectively, at EC5 and EC10, respectively | Niu et al. 2020 |
| Juncaceae | *Juncus effusus* | 5-10 dS·m^-1^, 8 weeks | Shoot DW reduction of 30%-49%; height and leaf area not affected at EC5; height, leaf area, and flower reductions of 13%-36%, 55%, 50%-89%, respectively | Sun and Palmer 2018 |
| Lamiaceae | *Agastache cana* | 4 dS·m^-1^, 10 weeks | Shoot DW and height not affected | Niu and Rodriguez 2006a |
| Lamiaceae | *Ajuga reptans* | 5 dS·m^-1^, 6 weeks | Shoot DW, leaf area, and flower reductions of 54%, 68%, and 100%, respectively | Wu et al. 2016c |
| Lamiaceae | *Caryopteris ×clandonensis* | 5-10 dS·m^-1^, 8 weeks | Shoot DW, height, and leaf area reductions of 20%-45%, 21%-39%, and 22%-63%, respectively | Wu et al. 2016a |
| Lamiaceae | *Coleus spp.* | ~19.3 dS·m^-1^, 1 weeks | Biomass, height, and leaf area reductions of 16%, 37%, and 36%, respectively | Kotagiri and Kolluru 2017 |
| Lamiaceae | *Lamium maculatum* | 5-10 dS·m^-1^, 6 weeks | Shoot DW, leaf area, flower reductions of 42%-68%, 61%-75%, and 49%-87%, respectively | Wu et al. 2016c |
| Lamiaceae | *Perovskia atriplicifolia* | 5-10 dS·m^-1^, 6 weeks | Shoot DW and leaf area reductions of 36%-78% and 67%, respectively | Wu et al. 2016c |
| Lamiaceae | *Poliomintha longiflora* | 5-10 dS·m^-1^, 6 weeks | Shoot DW and leaf area reductions of 62% and 69%, respectively | Wu et al. 2016c |
| Lamiaceae | *Rosmarinus officinalis* | 5.4 dS·m^-1^, 103 days | Shoot FW not affected | Niu et al. 2007 |
| Lamiaceae | *Salvia coccinea* | 2 dS·m^-1^, 10 weeks | Shoot DW not affected; significant reduction on height | Niu and Rodriguez 2006a |
| Lamiaceae | *Salvia farinacea* | 5-10 dS·m^-1^, 8 weeks | Shoot DW, height, and flower reductions of 12%-29%, 15%-25%, and 18%-29%, respectively | Sun et al. 2015a |
| Lamiaceae | *Salvia leucantha* | 5-10 dS·m^-1^, 8 weeks | Shoot DW, height, and flower reductions of 23%-55%, 13%-61%, and 42%-89%, respectively | Sun et al. 2015a |
| Lamiaceae | *Salvia splendens* | ~5.2 dS·m^-1^, 5 weeks | Significant reduction on DW and height | Villarino and Mattson 2011 |
| Lamiaceae | *Scutellaria suffrutescens* | 5 dS·m^-1^, 6 weeks | Shoot DW, leaf area, flower reductions of 70%, 87%, and 83%, respectively | Wu et al. 2016c |
| Lamiaceae | *Stachys coccinea* | 5-10 dS·m^-1^, 6 weeks | Shoot DW, leaf area, and flower reductions of 10%-56%, 11%-43%, and 27%-73%, respectively | Wu et al. 2016c |
| Lamiaceae | *Teucrium chamaedrys* | 3.2-12 dS·m^-1^, 12 weeks | Shoot DW reductions of 35%-65% | Niu and Rodriguez 2006b |
| Lythraceae | *Cuphea hyssopifolia* | 5-10 dS·m^-1^, 8 weeks | Shoot DW, height, and leaf area reductions of 15%-49%, 5%-20%, and 8%-89%, respectively | Wu et al. 2016a |
| Malvaceae | *Hibiscus syriacus* | 6.5 dS·m^-1^, 11 weeks | Shoot DW not affected | Chen et al. 2019a |
| Malvaceae | *Hibiscus syriacus* | 5-10 dS·m^-1^, 8 weeks | Shoot DW, height, and leaf area reductions of 25%-61%, 7%-25%, and 7%-70%, respectively | Liu et al. 2017 |
| Malvaceae | *Malvaviscus arboreus* | 5-10 dS·m^-1^, 8 weeks | Shoot DW, height, and flower reductions of 10%-43%, 20%-42%, and 300% respectively | Sun et al. 2015a |
| Malvaceae | *Pavonia lasiopetala* | 5-10 dS·m^-1^, 8 weeks | Shoot DW, height, and leaf area reductions of 44%-58%, 4%-8%, and 43%-68%, respectively | Wu et al. 2016a |
| Oleaceae | *Forsythia ×intermedia* | 5-10 dS·m^-1^, 8 weeks | Shoot DW, height, and leaf area 55%-77%, 22%-55%, and 58%-89%, respectively | Liu et al. 2017 |
| Onagraceae | *Fuchsia hybrida* | ~5.2 dS·m^-1^, 5 weeks | Significant reductions on DW and height | Villarino and Mattson 2011 |
| Papaveracea | *Glaucium flavum* | ~38.6 dS·m^-1^, 60 days | Growth rate reduction of 95% | Cambrollé et al. 2011 |
| Plantaginaceae | *Angelonia angustifolia* | 7.4 dS·m^-1^, 122 days | Shoot DW reduction of 50% | Niu et al. 2010b |
| Plantaginaceae | *Antirrhinum majus* | ~5.2 dS·m^-1^, 76 days | Height, leaf area, #flowers, and #leaves reductions of 26%, 63%, 16%, and 8%, respectively; shoot DW not affected | El-Attar 2017 |
| Plantaginaceae | *Antirrhinum majus* | 14 dS·m^-1^, 42 days | Significant reduction on height and flower weight | Carter and Grieve 2008 |
| Plantaginaceae | *Bacopa monneiri* | ~7.4 dS·m^-1^, 20 days | Shoot DW reduction of 54% | Khaliel et al. 2011 |
| Plantaginaceae | *Penstemon barbatus* | 2.5-10 dS·m^-1^, 8 weeks | Shoot DW reduction of 7%-18%; height and leaf area reductions of 84%-94% and 87% from EC7.5 to EC10 | Paudel and Sun 2024 |
| Plantaginaceae | *Penstemon davidsonii* | 2.5-10 dS·m^-1^, 8 weeks | Shoot DW not affected; leaf area reduction of 24% | Nepal et al. 2024 |
| Plantaginaceae | *Penstemon heterophyllus* | 2.5-10 dS·m^-1^, 8 weeks | Shoot DW and leaf area reductions of 53% and 72%, respectively | Nepal et al. 2024 |
| Plantaginaceae | *Penstemon strictus* | 2.5-10 dS·m^-1^, 8 weeks | Shoot DW reduction of 13%-31%; height not affected; leaf area reduction of 69%-92% at EC7.5 to EC10 | Paudel and Sun 2024 |
| Plumbaginaceae | *Ceratostigma plumbaginoides* | 3.2-6.4 dS·m^-1^, 12 weeks | Shoot DW reductions of 47%-63% | Niu and Rodriguez 2006b |
| Poaceae | *Andropogon ternarius* | 10 dS·m^-1^, 95 days | Shoot DW, height, and leaf area reductions of 37%-79%, 27%-45%, and 32%-76%, respectively | Xing et al. 2021 |
| Poaceae | *Bouteloua gracilis* | 5-10 dS·m^-1^, 18 weeks | Height reduction of 13%-36%; leaf area not affected at EC5 | Sun and Palmer 2018 |
| Poaceae | *Calamagrostis ×acutiflora* | 5-10 dS·m^-1^, 95 days | Shoot DW, height, and leaf area reductions of 37%-79%, 16%-45%, and 32%-76%, respectively | Xing et al. 2021 |
| Poaceae | *Chasmanthium latifolium* | 5-10 dS·m^-1^, 18 weeks | Shoot DW reduction of 55% at EC5; height and leaf area reductions of 13%-36% and 52%, respectively, at EC10 | Sun and Palmer 2018 |
| Poaceae | *Eragrostis spectabilis* | 5-10 dS·m^-1^, 65 days | Shoot DW, height, and leaf area reductions of 25%-46%, 15%, and 22%-47%, respectively | Wang et al. 2019b |
| Poaceae | *Festuca glauca* | 10 dS·m^-1^, 95 days | Shoot DW, height, and leaf area reductions of 37%-79%, 14%, and 32%-76% | Xing et al. 2021 |
| Poaceae | *Leymus arenarius* | 5-10 dS·m^-1^, 18 weeks | Shoot DW reduction of 19% at EC10; height and leaf area not affected | Sun and Palmer 2018 |
| Poaceae | *Miscanthus sinensis* | 10 dS·m^-1^, 65 days | Shoot DW, height, and leaf area reductions of 25%-46%, 15%, and 22%-47%, respectively | Wang et al. 2019b |
| Poaceae | *Muhlenbergia capillaris* | 10 dS·m^-1^, 18 weeks | Shoot DW, height and leaf area reductions of 29%, 13%-36% and 46%, repectively | Sun and Palmer 2018 |
| Poaceae | *Panicum virgatum* | 10 dS·m^-1^, 65 days | Shoot DW, height, and leaf area reductions of 25%-46%, 15%, and 22%-47%, respectively | Wang et al. 2019b |
| Poaceae | *Panicum virgatum* | 10 dS·m^-1^, 4 weeks | Shoot DW, height, and leaf area reductions of 28%-63%, 22%-35%, and 51%-65%, respectively | Sun et al. 2018a |
| Poaceae | *Pennisetum alopecuroides* | 10 dS·m^-1^, 18 weeks | Shoor DW, height, and flower reductions of 41%, 13-36%, and 48%, respectively | Sun and Palmer 2018 |
| Poaceae | *Pennisetum americanum* | 20 dS·m^-1^, 4 weeks | Shoot DW and height reductions of 40%-50% and 30%-50%, respectively | Ashraf and Mcneilly 1987 |
| Poaceae | *Schizachyrium scoparium* | 10 dS·m^-1^, 65 days | Shoot DW, height, and leaf area reductions of 25%-46%, 15%, and 22%-47%, respectively | Wang et al. 2019b |
| Poaceae | *Sporobolus heterolepis* | 5-10 dS·m^-1^, 95 days | Shoot DW and leaf area reductions of 37%-79% and 32%-76%, respectively | Xing et al. 2021 |
| Poaceae | *Zoysia matrella* | 10 dS·m^-1^, 8 weeks | Shoot DW not affected; leaf area reduction of 10% | Hooks et al. 2022 |
| Poaceae | *Zoysia minima* | 10 dS·m^-1^, 8 weeks | Leaf area reduction of 27% | Hooks et al. 2022 |
| Poaceae | *Zoysia japonica* | 10 dS·m^-1^, 8 weeks | Leaf area reduction of 10% | Hooks et al. 2022 |
| Polemoniaceae | *Phlox paniculata* | 5-10 dS·m^-1^, 8 weeks | Shoot DW and height reductions of 21%-57% and 9%-37%, respectively | Sun et al. 2015a |
| Portulacaceae | *Portulaca grandiflora* | 3.2 dS·m^-1^, 3 months | Height, #shoots, and #leaves reductions of 8%, 6%, and 3%, respectively | Gupta et al. 2018 |
| Portulacaceae | *Portulaca grandiflora* | 4.5 dS·m^-1^, 8 weeks | Shoot DW and height reductions of 10% and 16%, respectively; flowers not affected | Devitt and Morris 1987 |
| Ranunculaceae | *Anemone coronaria* | 4.5 dS·m^-1^, 8 weeks | Shoot DW not affected | Rauter et al. 2021 |
| Ranunculaceae | *Aquilegia canadensis* | 5-10 dS·m^-1^, 8 weeks | Shoot DW, height, and leaf area reductions of 50%, 22%-74%, and 30%, respectively | Wu et al. 2016c |
| Ranunculaceae | *Ranunculus asiaticus* | 5.5 dS·m^-1^, 8 weeks | Shoot DW not affected | Rauter et al. 2021 |
| Ranunculaceae | *Ranunculus asiaticus* | 6 dS·m^-1^, 88 days | Shoot DW and flower reductions of 84% and 46%, respectively | Valdez-Aguilar et al. 2009 |
| Ranunculaceae | *Ranunculus acris* | 5.8 dS·m^-1^, 48 days | Significant reduction on DW and leaf area | Wala et al. 2023 |
| Ranunculaceae | *Ranunculus sceleratus* | 15.6 dS·m^-1^, 5 weeks | Biomass reduction of 44% | Ievinsh et al. 2022 |
| Rosaceae | *Cercocarpus ledifolius* | 5 dS·m^-1^, 8 weeks | Leaf area reduction of 26% | Paudel and Sun 2023 |
| Rosaceae | *Cercocarpus montanus* | 10 dS·m^-1^, 8 weeks | Leaf area reduction of 44% | Paudel and Sun 2023 |
| Rosaceae | *Chaenomeles speciosa* | 5 dS·m^-1^, 8 weeks | Shoot DW, height, and leaf area reductions of 57%-64%, 5%-34%, and 69%-72%, respectively | Liu et al. 2017 |
| Rosaceae | *Physocarpus opulifolius* | 4.6-6.5 dS·m^-1^, 11 weeks | Biomass reduction of 50% | Chen et al. 2019a |
| Rosaceae | *Spiraea japonica* | 3-6 dS·m^-1^, 8 weeks | Up to 65% reduction on shoot DW; leaf area reduction of 21%-49% | Wang et al. 2019a |
| Rosaceae | *Spiraea japonica* | 5.4-6.5 dS·m^-1^, 11 weeks | Biomass reduction of 50% | Chen et al. 2019a |
| Rosaceae | *Rosa fortuniana* | 9 dS·m^-1^, 15 weeks | Shoot DW reduction of 33% | Niu et al. 2008 |
| Rosaceae | *Rosa ×hybrida* | 4-8 dS·m^-1^, 54 days | Shoot DW reductions of 28%-69%; flower reductions of 42%-77% | Cai et al. 2014b |
| Rosaceae | *Rosa multiflora* | 9 dS·m^-1^, 15 weeks | Shoot DW reduction of 55% | Niu et al. 2008 |
| Rosaceae | *Rosa odorata* | 9 dS·m^-1^, 15 weeks | Shoot DW reduction of 49% | Niu et al. 2008 |
| Rosaceae | *Rosa spp.* | 6.4 dS·m^-1^, 7 weeks | Shoot DW reductions of 32%-42%; flower reductions of 48%-77% | Niu et al. 2013 |
| Rosaceae | *Rosa spp.* | 6.4 dS·m^-1^, 10 weeks | Shoot DW reductions of 30%-75%; flower reductions of 52%-80% | Niu et al. 2013 |
| Rosaceae | *Rosa spp.* | 10 dS·m^-1^, 43 days | Shoot DW and flower reductions of 18%-82% and 8%-62%, respectively | Cai et al. 2014a |
| Solanaceae | *Capsicum annuum* | 8.1 dS·m^-1^, 57 days | Shoot DW reduction of 42% | Niu et al. 2012a |
| Solanaceae | *Capsicum annuum* | 4.1 dS·m^-1^, 74 days | DW reduction of 22%-92% | Niu et al. 2010c |
| Solanaceae | *Cestrum spp.* | 10 dS·m^-1^, 8 weeks | Shoot DW, height, and leaf area reductions of 18%-40%, 8%-28%, and 24%-50%, respectively | Wu et al. 2016a |
| Solanaceae | *Nicotiana rustica* | 6.4 dS·m^-1^, 100 days | Significant reduction on shoot DW and height | Cusido et al. 1987 |
| Solanaceae | *Nicotiana tabacum* | 15 dS·m^-1^, 77 days | Height, leaf DW, and #leaves reductions of 56%, 65%, and 53%, respectively | Tok and Temizel 2022 |
| Solanaceae | *Petunia spp.* | ~5.2 dS·m^-1^, 5 weeks | Significant reductions of DW and height | Villarino and Mattson 2011 |
| Solanaceae | *Petunia hybrid* | 11 dS·m^-1^, 45 days | Height and #leaves reductions of 42% and 60%, respectively | Wahocho et al. 2023 |
| Solanaceae | *Petunia hybrid* | 12.5 dS·m^-1^, 30 days | DW, shoot DW, and #leaves reductions of 27%, 18%, and 32%, respectively; flowers not affected | Fornes et al. 2007 |
| Verbenaceae | *Glandularia canadensis* | 3.2-5.4 dS·m^-1^, 103 days | Shoot FW not affected | Niu et al. 2007 |
| Verbenaceae | *Glandularia ×hybrida* | 3.2-5.4 dS·m^-1^, 103 days | Shoot FW reductions of 44%-59% | Niu et al. 2007 |
| Verbenaceae | *Lantana camara* | 5.1 dS·m^-1^, 175 days | DW and leaf area reductions of 37% and 50%, respectively | Bañón et al. 2011 |
| Verbenaceae | *Lantana montevidensis* | 2.2 dS·m^-1^, 180days | DW, #flower, #shoots, and diameter reductions of 22%, 34%, 16%, and 105%, respectively; leaf area increase of 13% | Cristiano et al. 2018 |
| Verbenaceae | *Lantana montevidensis* | 3.2-5.4 dS·m^-1^, 103 days | Shoot FW reductions of 41%-75% | Niu et al. 2007 |
| Verbenaceae | *Lantana ×hybrida* | 5.4 dS·m^-1^, 103 days | Shoot FW not affected | Niu et al. 2007 |
| Verbenaceae | *Verbena ×hybrida* | 5-10 dS·m^-1^, 8 weeks | Shoot DW, height, flower reductions of 11%-21%, 7%-30%, and 15%, respectively | Sun et al. 2015a |
| Verbenaceae | *Verbena macdougalii* | 5.4 dS·m^-1^, 103 days | Shoot FW not affected | Niu et al. 2007 |
| Verbenaceae | *Verbena officinallis* | 3.9 dS·m^-1^, 12 weeks | DW, height, #flowers, and #shoots reductions of 28%, 44%, 63%, and 34%, respectively | Çiçek and Yücedağ 2023 |
| Verbenaceae | *Verbena officinallis* | 8.5 dS·m^-1^, 30 days | Significant reduction on biomass and height | Chrysargyris et al. 2021 |
| Violaceae | *Violax ×Wittrockiana* | 3.1 dS·m^-1^, 8 weeks | DW and height reductions of 79% and 82%, respectively | Kuehny and Morales 1998 |
| Violaceae | *Viola ×Wittrockiana* | 10.9 dS·m^-1^, 8 weeks | Flower and #leaves reductions of 33% and 15%, respectively; height increase 9% | PUŠIĆ et al. 2019 |
| Vitaceae | *Parthenocissus quinquefolia* | 5-10 dS·m^-1^, 8 weeks | Shoot DW, height, and leaf area reductions of 47%-65%, 45%-87%, and 8%-15%, respectively | Liu et al. 2017 |

^i^ the electricity conductivity (EC) of saline irrigation

^ii^ the duration of saline irrigation
